# A scalable adaptive strategy for influence maximization in temporal social networks via vulture based meta heuristic

**DOI:** 10.1038/s41598-025-24746-6

**Published:** 2025-11-20

**Authors:** Linian Liu, Binrong Huang, Shouliang Lai

**Affiliations:** 1 Hanyang University, Seoul, 04763 Korea; 2 College of Packaging Design and Art, Hunan University of Technology, Zhuzhou, 412000 Hunan China

**Keywords:** Dynamic social networks, Influence maximization, Vulture adaptive algorithm, Meta-Heuristic optimization, Scalability, Engineering, Mathematics and computing

## Abstract

Over the past decade, social networks have become vital forums for engagement, opinion formation, and information dissemination in areas such as marketing, policymaking, and public health. Identifying key individuals within these networks poses a considerable challenge, especially due to their dynamic nature and broad extent. This article introduces the Adaptive Dynamic Vulture Algorithm (ADVA) as a novel Meta-Heuristic method for improving influence in dynamic social networks. This methodology achieves an optimal balance between exploration and exploitation by prioritizing adaptation to temporal variations in networks and scalability, two aspects often neglected in previous studies. ADVA maintains its efficiency by adaptively adjusting the search methodology in response to changes in network design, such as edge density and node connectivity. The main challenge of this strategy is the computational complexity resulting from the handling of dynamic data. While pruning and indexing approaches alleviate this problem to a degree, they nonetheless result in longer execution times compared to certain alternative solutions. Evaluations on benchmark datasets, such as Stack Overflow and Wiki Talk, demonstrate that ADVA improves penetration by 15% on Stack Overflow and 20% on Wiki Talk compared to prior techniques, while maintaining scalability in large networks. This advantage is attributed to its adaptive techniques and multi-stage optimization; nonetheless, the extended execution time (e.g., 4800 s for a seed size of 60 on Stack Overflow) indicates a need for improvements in computing efficiency.

## Introduction

 Social media platforms are becoming widely used infrastructures for population-scale coordination, communication, and information dissemination. In areas including marketing, public health, and crisis response, they influence collective action, consumer behavior, and attitudes beyond casual encounters^1^. Real-world interactions are intrinsically temporal; nodes and edges emerge, vanish, and rewire over time, resulting in sequences of time-stamped contacts rather than a single, stable topology, despite the fact that a large portion of the early study viewed these systems as static graphs. The graph of a temporal (or dynamic) social network is typically represented as a time-stamped edge stream or as an ordered sequence of snapshots {$\:G\left(1\right),\:G\left(2\right),\:\dots\:,\:G\left(T\right)\}$. In these networks, the speed and viability of information flow rely on when ties exist rather than just whether they do.

Applications ranging from viral advertising to rumor containment and public-information campaigns are supported by influence, which is the capacity of a fraction of users to set off significant downstream cascades, inside such networks. A seed set of size k that maximizes predicted diffusion under a contagion model is required for the associated computing job, influence maximization (IM). The submodularity of the problem under standard models was proved by classical IM studies, which also produced greedy approximations with (1–1/e) guarantees. However, previous studies mostly assumed static graphs and relied on costly Monte Carlo estimation^1–3^. Recalculating seeds from start becomes impractical as networks expand and change, and the efficacy of seeds chosen from out-of-date snapshots rapidly deteriorates.

Beyond scale, temporal situations present new difficulties^4^. First, since neglecting timing can overstate the influence of a seed set, diffusion in temporal graphs must obey time-ordering. Second, techniques that rely on fixed structural assumptions deteriorate rapidly because non-stationarity causes centrality and community structures to change as activity rises and falls^5,6^. Third, integrity of influence estimation and computational efficiency are trade-offs: pruning lowers search costs but may unintentionally remove nodes that quickly become crucial^7^. Lastly, assessing diffusion in temporal networks necessitates striking a balance between exploitation using existing loci of influence and exploration adjusting to novel structural conditions. When combined, these problems significantly increase the complexity of influence maximization in temporal networks compared to static ones^8^.

Practical applications highlight the significance of tackling these issues. In marketing, companies look for customers whose impact is at its highest around specific times, such new product launches or seasonal advertising campaigns^9^. During outbreaks, when network contacts change in real time, public health officials must identify which people or communities are best suited to spread critical information. Similarly, political campaigns need flexible tactics to track and react to changing patterns of influence in various areas. Strategies that lack temporal awareness run the danger of misallocating or underestimating resources, which could result in ineffective or even detrimental effects^10^.

Current methods only offer limited answers. Despite having a theoretical foundation, greedy algorithms become unaffordable when scaled to millions of nodes and edges. Although they lower processing cost, structural heuristics like degree or PageRank centrality frequently oversimplify influence pathways and miss time-sensitive effects^11^. Through iterative exploration of the search space, meta-heuristic techniques such as Genetic Algorithms (GA), Particle Swarm Optimization (PSO), and Ant Colony Optimization (ACO) provide enhanced scalability. Nevertheless, the majority lacked methods to adjust to the temporal evolution of real-world social networks because they were created for static environments^12–14^. Though they require significant training resources and frequently have trouble generalizing when network structure changes quickly, graph embedding and deep learning-based models exhibit promise. Few algorithms are both computationally scalable and temporally aware, according to this survey of approaches^15^.

The creation of techniques that specifically combine temporal flexibility and effective optimization is driven by these gaps. Population-based meta-heuristics, which strike a balance between exploration and exploitation while preserving the diversity of potential solutions, provide a promising avenue. Vulture-inspired algorithms, which mimic the adaptive foraging behavior of vultures to alternate between intensification and diversification, have become popular approaches for solving complex optimization issues within this family. For instance, by dynamically modifying search operators, the African Vulture Optimization Algorithm (AVOA) has excellent performance across static benchmarks^16,17^. However, techniques to manage temporal variability in data streams were not included in its initial design.

This line of inquiry is continued by our suggested approach, the ADVA, which incorporates temporal awareness straight into the optimization procedure. The exploration-exploitation trade-off is conceptually carried over from AVOA to ADVA, although it undergoes three significant modifications. In order to adjust parameters like exploration rate and centrality weighting online based on structural indications like edge density and degree distribution, it first presents data-driven adaptive controls. Second, in order to improve scalability without compromising accuracy, ADVA incorporates reachability-based pruning and indexing algorithms to concentrate the search on nodes with the highest potential for near-term influence. Third, it assesses diffusion across temporal snapshot sequences using the Independent Cascade model, guaranteeing that influence estimates take into account the time-sensitive nature of network connectedness. Through these improvements, ADVA is intended to be a temporal and influence-aware adaptation that works well with vast, dynamic social graphs rather than a direct implementation of AVOA.

In conclusion, a distinct set of research gaps the insufficiency of static models, the lack of temporal adaptability in the majority of meta-heuristics, and the conflict between diffusion fidelity and computational efficiency motivated the invention of ADVA. This study attempts to offer a technique that is more in line with the realities of temporal social networks by placing ADVA within the family of vulture-based meta-heuristics as well as the literature on influence maximization.

This is how the rest of the paper is structured. The related work on static and dynamic influence maximization is reviewed in Sect. "Related works" The design of ADVA, including adaptive parameterization, pruning techniques, and integration with diffusion models, is covered in length in Sect. "Adaptive dynamic Vulture algorithm (ADVA)". Experimental results on benchmark datasets are presented in Sect. "Evaluation and simulation", and ramifications and future research objectives are discussed in Sect. "Conclusion".

## Related works

The purpose of IM, which has long been acknowledged as a crucial issue in the field of social networks, is to find a small subset of important nodes (the “seed set”) that have the capacity to start the greatest potential chain reaction of influence. Numerous algorithmic approaches, such as community-aware tactics, meta-heuristic optimization models, and conventional heuristics, have been drawn to this topic. This section presents a summary of a number of well-known algorithms, such as IMBC, DBATM, MFA, IWHGAO, and HIGHDEG, that served as baselines in our experimental study.

Effective seed selection and time efficiency are two of the main issues in instant messaging that are addressed by the IMBC (Influence Maximization Based on Community Structure) algorithm^18^. It improves the quality of seed selection by adjusting node scores according to the Rich-Club coefficient and introducing an optimal pruning strategy that uses minimum dominating sets to lower the computational cost. IMBC enhances scalability and influence propagation in large-scale networks by fusing score reweighting with community structure awareness.

By converting the continuous BAT algorithm into a discrete version appropriate for instant messaging, DBATM (Discrete BAT Modified)^19^ is the result of additional study. DBATM offers a hybrid model for choosing the best seeds by combining discrete swarm intelligence concepts with rank-based methods like PageRank and NodeRank. Its efficacy across several datasets was shown in experiments in^19^, underscoring its potential for discrete influence optimization.

In order to maximize influence in social networks, the Moth–Flame Algorithm (MFA), which was first created for continuous optimization, was modified in^20^. The main concept is to successfully explore the solution space by simulating a flame-tracking system. The algorithm maximizes a fitness function obtained from the diffusion process in order to locate seed nodes with high influence potential. MFA is especially valued for its capacity to sustain investigation and steer clear of local optima.

In order to increase search variety and convergence speed, the IWHGAO (Improved Wild Horse Genetic Algorithm–Based Optimization)^21^ presents a hybrid approach that combines genetic algorithms with wild horse movement tactics. IWHGAO, which was created especially for community-based social networks, strikes a balance between intensification and diversification, allowing precise community identification and seed selection in situations involving influence propagation.

HIGHDEG^22^, on the other hand, adopts a traditional strategy of choosing nodes with the highest degrees as potential seed candidates. Despite being quick and easy to compute, HIGHDEG ignores community dynamics and temporal change. Remarkably^22^, also suggests a multi-objective extension to instant messaging with the goal of minimizing the initial node count while optimizing influence diffusion, which is crucial for applications that are cost-sensitive.

These algorithms include a wide range of instant messaging tactics, including heuristic, structural, and nature-inspired meta-heuristics. Each presents different trade-offs between diffusion performance, complexity, and efficiency. In this study, we empirically illustrate the benefits of our suggested ADVA algorithm in dynamic, temporal networks by comparing it to these baselines.

### Problem statement

Social networks are essential for marketing, influencing public opinion, and crisis management because of their exponential expansion, which has completely changed the way information is produced, shared, and consumed^23^. The problem of IM, or finding a selection of powerful nodes that can maximize the spread of information, gets more difficult as these networks develop. Instant messaging has shown promise in areas including viral marketing, electoral campaigns, and product uptake. The time aspect of real-world networks is often overlooked by traditional instant messaging techniques, which typically work on static graphs. Diffusion processes can be more realistically modeled thanks to temporal networks, which are sequences of time-stamped snapshots $\:\left\{{\text{G}}_{1},\:{\text{G}}_{2},\dots\:,\:{\text{G}}_{T}\right\}$ that capture changing user interactions^24^. Temporal Influence Maximization (IMT) seeks to dynamically choose seed nodes in these situations while taking user behavior and topological variations into consideration. Several challenges make IMT considerably more difficult than its static counterpart:

- Computational complexity: Recomputing seeds per snapshot is often infeasible, especially using Monte Carlo simulations^23^.
- Bias from static metrics: Static measures such as PageRank or degree often fail to capture time-sensitive influence potential^25^.

Greedy algorithms like CELF and IMM are examples of classic solutions; they provide (1–1/e) approximation guarantees but have scaling problems. Although heuristic techniques like PageRank and High Degree minimize computation, they are susceptible to network dynamics. For more reliable influence estimate, recent hybrid techniques like the MKS algorithm^24^ make use of both local and global node properties.

Meta-heuristic algorithms such as GA and PSO have been investigated for instant messaging in order to get around the drawbacks of greedy methods. These techniques scale more effectively and adjust to network fluctuations by maintaining varied populations and searching through iterative refinements^26^. However, traditional GA and PSO are less resilient in dynamic networks because they lack temporal adaptation and frequently call for manual parameter adjustment.

For temporal influence analysis, more recent attempts use reinforcement learning and Graph Neural Networks (GNNs). For example, The adaptive technique is supplemented by an event-based adaptive fuzzy dynamic control framework for cascaded PDE-ODE systems with actuator failures^27^. Their solution decreases computational cost while preserving robustness by updating only when actuator requirements are met, corresponding with ADVA’s instantaneous adaptation and computationally conscious tuning in temporally non-stationary networks. In a similar vein, A safe fuzzy weight-based coordination control strategy for second-order heterogeneous multi-agent systems taught to modify online coordination weights via reinforcement learning is presented^28^. Their resistance against RL-induced disruptions and minimal human tuning allow parallel ADVA data-driven parameter adaption for flexible operation under time-varying interaction structures. While^29^ integrated user profiles with a multi-layer attention mechanism for privacy-preserving access restriction in social networks^30^, employed heterogeneous GNNs and R-GCN for relational clustering-based moralized radical content detection.

Adaptive influence techniques have also made use of reinforcement learning (RL). The dynamic negative emotion contagion model (NECM), which uses Deep Q-Networks (DQN) to adapt to changing sentiment spread, was first presented in^31^. In order to enhance early adopter selection in dynamic networks, GIMDRL, a deep reinforcement learning framework that integrates node embeddings from multiple GNNs, was proposed in a study^26^. Influence spread was studied in a paper^32^ using Stackelberg-Nash games with optimum control under time delays.

In addition to influence maximization, studies like^33^ looked into behavioral impacts like social crowding in mobile commerce, and^34^ used multi-relational graphs with heterophily and homophily disentanglement to create fraud detection models. In^35^, dynamic private opinion weights in community-based networks were used to model the Spiral of Silence theory.

Despite recent advances, three fundamental gaps still remain in the field of impact maximization. First, lack of adaptability: most classical and heuristic approaches are unable to automatically adjust parameters based on network structure or evolution. Second, lack of scalability: methods such as IMM and deep learning-based models are still computationally too expensive for large-scale and real-time applications. Third, lack of temporal awareness: static impact estimators ignore the effect of temporal order and accessibility over time.

To address these shortcomings, this paper introduces the ADVA, a scalable metaheuristic that integrates temporal pruning, adaptive parameter tuning based on structural signals, and temporal snapshot-aware propagation modeling. Aiming to create a dynamic balance between exploration and exploitation, ADVA offers superior performance capabilities over both classical and deep learning-based methods in diverse network environments.

Table 1 presents a comparison of different optimization techniques for influence maximization in social networks with the suggested ADVA method. The suggested ADVA approach facilitates the management of dynamic networks through adaptive pruning and snapshot-based optimization. In contrast to other metaheuristic methods that necessitate meticulous parameter optimization and intricate computations, ADVA employs a streamlined evolutionary algorithm that considers the temporal dynamics of the network.

**Table 1:** Comparison between different optimization methods for maximizing impact on social networks.

Ref.	Method	Graph Dynamics	Optimization Technique	Limitation
^18^	Partial payoff dominating set (NP-complete)	Static	MIP formulations	High complexity, lacks adaptability
^24^	Threshold-based dominating set selection	Static	Weighted thresholds	Fixed thresholds reduce flexibility
^19^	Discrete BAT algorithm for influence spread	Indirect	Bio-inspired metaheuristic	Requires extensive parameter tuning
^26^	Graph embedding with struc2vec + GNN	Dynamic	Embedding + deep learning model	Requires offline training and embedding computation
^20^	Moth-Flame Optimization (MFA) for influence maximization	Static	Metaheuristic swarm optimization	Sensitive to initial parameters
^29^	Richclub-based transfer and charismatic node identification	Static	Community-based heuristic	Limited to predefined high-degree clusters
^21^	Wild Horse Genetic Algorithm Optimization (IWHGAO)	Static	Hybrid evolutionary metaheuristic	Slower convergence on large-scale networks
ADVA (Our Method)	Adaptive pruning and snapshot-based temporal optimization (proposed)	Dynamic	Hybrid adaptive evolutionary model	Slightly higher runtime due to temporal tuning

## Adaptive dynamic Vulture algorithm (ADVA)

Social networks are intrinsically dynamic, with relationships and interactions always changing as users engage, disengage, or alter their associations. This dynamism presents a considerable problem for influence maximization, as static methods rapidly become obsolete, and computational complexity increases with network expansion. This article introduces the ADVA, a meta-heuristic approach aimed at effectively identifying influential nodes in dynamic social networks. ADVA utilizes adaptability and scalability, customizing its search methodology to temporal variations while reducing computational burden. By representing the network as a series of temporal snapshots, the approach guarantees that the seed set progresses in alignment with the network’s topology.

### Problem setting and Temporal modeling

A dynamic social network can be depicted as a sequence of graphs $\:\left\{{G}_{1},\:{G}_{2},\:\dots\:,\:{G}_{n}\right\}$, where each $\:G_{t}\:=\:\left(V_{t},\:E_{t}\right)$ encompasses the vertices $\:V_{t}$ and edges $\:E_{t}$ that are active at time $\:t$^34^.

**Fig. 1:**
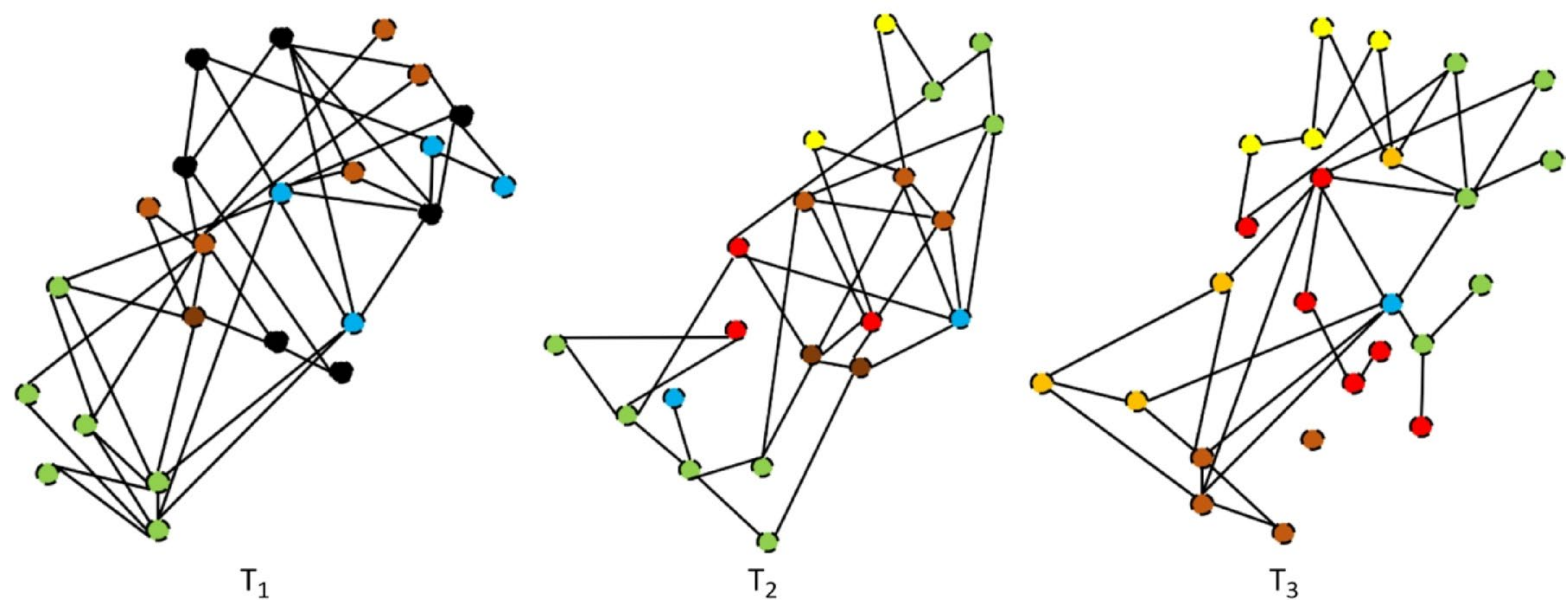
An example of a temporal social network represented as three snapshots $\:{\mathbf{T}}_{1},\:{\mathbf{T}}_{2},\:{\mathbf{T}}_{3}$ showing the evolution of nodes and edges over time.

This dynamic social network is depicted as a series of temporal snapshots $\:{\mathbf{T}}_{1},\:{\mathbf{T}}_{2},\:{\mathbf{T}}_{3}$, as seen in Fig. 1. The collection of active nodes and their interactions at a specific moment are captured in each snapshot. As time passes, the connectivity patterns shift and some nodes and edges appear or go. The necessity for algorithms like ADVA that can adaptively update the seed set as the network structure changes is highlighted by this temporal evolution. Influential candidates, for instance, are concentrated in the lower portion of the graph in $\:{\mathbf{T}}_{1}$, whereas new connections appear in the upper portion of $\:{\varvec{T}}_{2}$ and $\:{\varvec{T}}_{3}$, potentially influencing different communities. Therefore, Fig. 1 is an example of why social networks should be modeled as temporal graphs as opposed to static structures.

The ADVA framework functions in two principal phases: startup and iterative refining. It first creates a candidate population by indexing accessible vertices from each node in $\:G_{t}$, thereby diminishing the search space by around 40% using pruning methods. This measure improves efficiency while preserving essential influencers. ADVA subsequently employs a vulture-inspired dynamic search that adjusts the exploration rate and the centrality weights in response to real-time network measurements (e.g., edge density and degree variations). The adaptive selection strategy minimizes latency in on-chip networks and offers an efficiency-oriented method that facilitates computational pruning of ADVA in dynamic networks^35,36^.

### Adaptive Meta-Heuristic framework

Algorithm 1 delineates the fundamental architecture of the Adaptive Meta-Heuristic Framework, functioning as a comprehensive blueprint for enhancing influence maximization in dynamic social networks. The input consists of a dynamic network $\:G$, time intervals $\:T$, seed size $\:k$, and influence probability $\:\lambda\:$, resulting in a seed set $\:{S}_{t}$ for each time step. The approach commences with the initialization of a candidate population $\:P$, which is progressively enhanced through a synthesis of local and global search methodologies^37^. Local search improves specific solutions, but global search incorporates wider network insights, amalgamating results to identify the top-k solutions according to a fitness function related to influence dissemination. This iterative refinement persists until a stopping criterion, such as convergence or a maximum iteration threshold, is achieved, with updates governed by a diffusion model. The versatility of this architecture and its dual-search methodology offer a versatile basis for ADVA, allowing it to effectively manage the changing topology of social networks.

We first present the high-level adaptive meta-heuristic framework that governs population initialization, local/global search, and fitness-guided selection.

**Algorithm 1:**
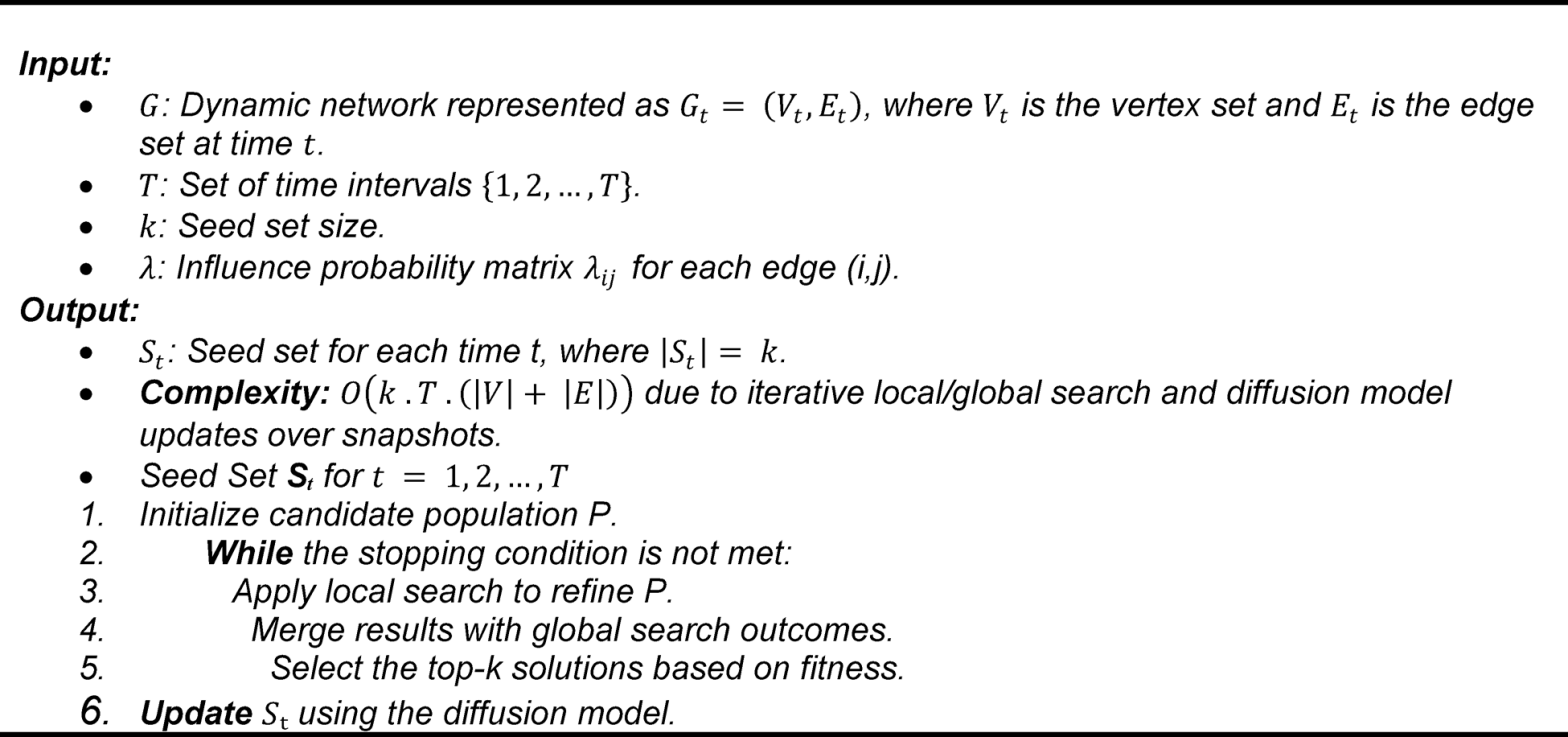
Adaptive Meta-Heuristic Framework.

### Operational framework

We then describe the operational pipeline executed at each snapshot to construct candidate sets via reachability, apply ADVA, and store $\:{S}_{t}$ across time.

Algorithm 2 delineates the fundamental operational framework of the ADVA, specifically designed for influence maximization over temporal snapshots. It accepts a dynamic network $\:G$, time intervals $\:T$, seed size $\:k$, and influence probability $\:\lambda\:$, producing an optimized seed set $\:{S}_{t}$ for each time $\:t$^38^. For each interval, it retrieves the snapshot $\:{G}_{t}$ and computes reachable nodes from each vertex v, pruning and indexing them into a candidate set $\:{C}_{t}$ to mitigate computational burden. ADVA subsequently employs its adaptive vulture-inspired optimization on $\:{C}_{t}$, resulting in $\:{S}_{t}$, which is retained for the subsequent iteration. This approach utilizes temporal continuity by relying on previous snapshots, guaranteeing the seed set adapts with the network.

**Algorithm 2:**
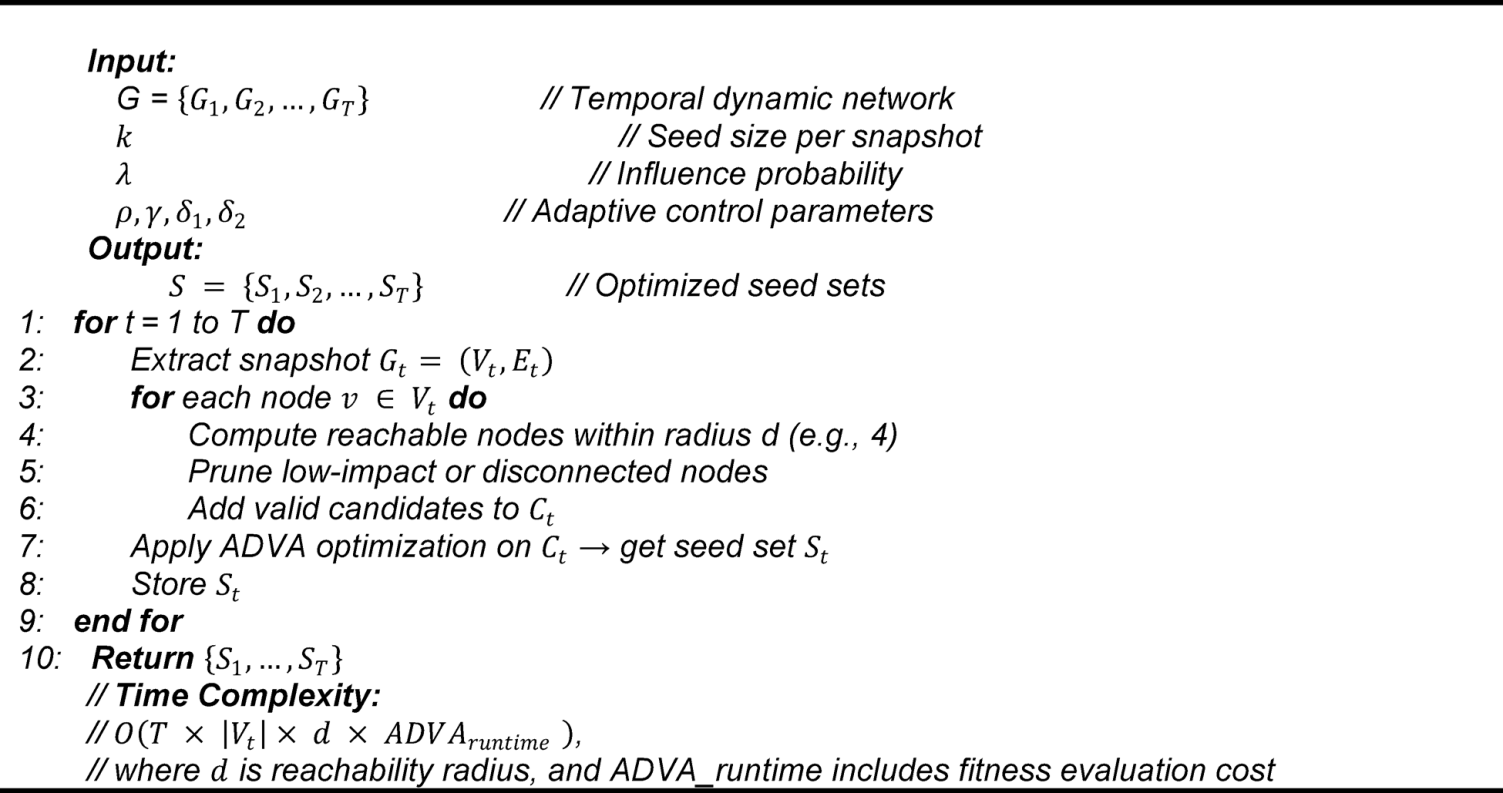
ADVA Core Process.

### Preliminary definitions and notations

Here, $\:{\lambda\:}_{ij}\in\:\left[\text{0,1}\right]$ denotes the influence probability of activation from node $\:i$ to node$\:\:j$, $\:\rho\:$ represents the edge density of the snapshot, $\:\gamma\:\:$is the adaptive exploration rate, and $\:{{\updelta\:}}_{1}$, $\:{{\updelta\:}}_{2}$ are exploitation thresholds controlling the merging intensity during the optimization process. The notation $\:a_{t}\left(S_{t}\right)$ refers to the expected influence spread at time $\:t$ for a given seed set $\:{S}_{t}$.

*Temporal Social Network.* We model a dynamic network as an ordered sequence of snapshots $\:\left\{{G}_{1},\:{G}_{2},\:\dots\:,\:{G}_{t}\right\}$, where each snapshot $\:G_{t}\:=\:\left(V_{t},\:E_{t}\right)$ represents the active vertex set $\:{V}_{t}$​ and edge set $\:{E}_{t}$ at time $\:t$. Paths used for diffusion must be *time-respecting*, meaning edges must occur in non-decreasing time order.

*Influence Maximization (IM).* Given a budget $\:k$, at each time $\:t$, we select a seed set $\:St\subseteq\:{V}_{t}$​ with $\:\left|{S}_{t}\right|=k\:$ to maximize the expected number of activated nodes under a specified diffusion model^39^.

*Independent Cascade (IC).* For a directed edge $\:\left(i,j\right)\in\:{E}_{t}$​, activation succeeds with probability $\:{\lambda\:}_{ij}\in\:\left[\text{0,1}\right]$. Each newly activated node has a single opportunity to activate each of its inactive neighbors in the next discrete time step. The diffusion process at time $\:t$ is evaluated on $\:{G}_{t}$, and the expected spread is denoted by $\:{a}_{t}\left({S}_{t}\right)$.

*Reachability Radius and Candidate Set.* To enhance scalability, we define a snapshot-specific candidate set $\:{C}_{t}\subseteq\:{V}_{t}$ based on reachability within a bounded radius from high-potential vertices^40^. This approach preserves time-respecting paths while pruning regions with low impact.

*Adaptive Signals.* Let $\:{\rho\:}_{t}=\left|{E}_{t}\right|/\left|{V}_{t}\right|\left(\right|{V}_{t}|-\:1)\:\:$​ denote the edge density of snapshot $\:{G}_{t}$. Additionally, let $\:de{g}_{t}\left(i\right),\:outde{g}_{t}\left(i\right)$, and $\:{B}_{t}\left(i\right)$ represent the degree, out-degree, and approximate betweenness centrality, respectively, for node $\:i$ in $\:{G}_{t}$. These statistics guide the online tuning of the exploration rate and centrality weighting in the ADVA algorithm.

### Pruning and indexing

**Fig. 2:**
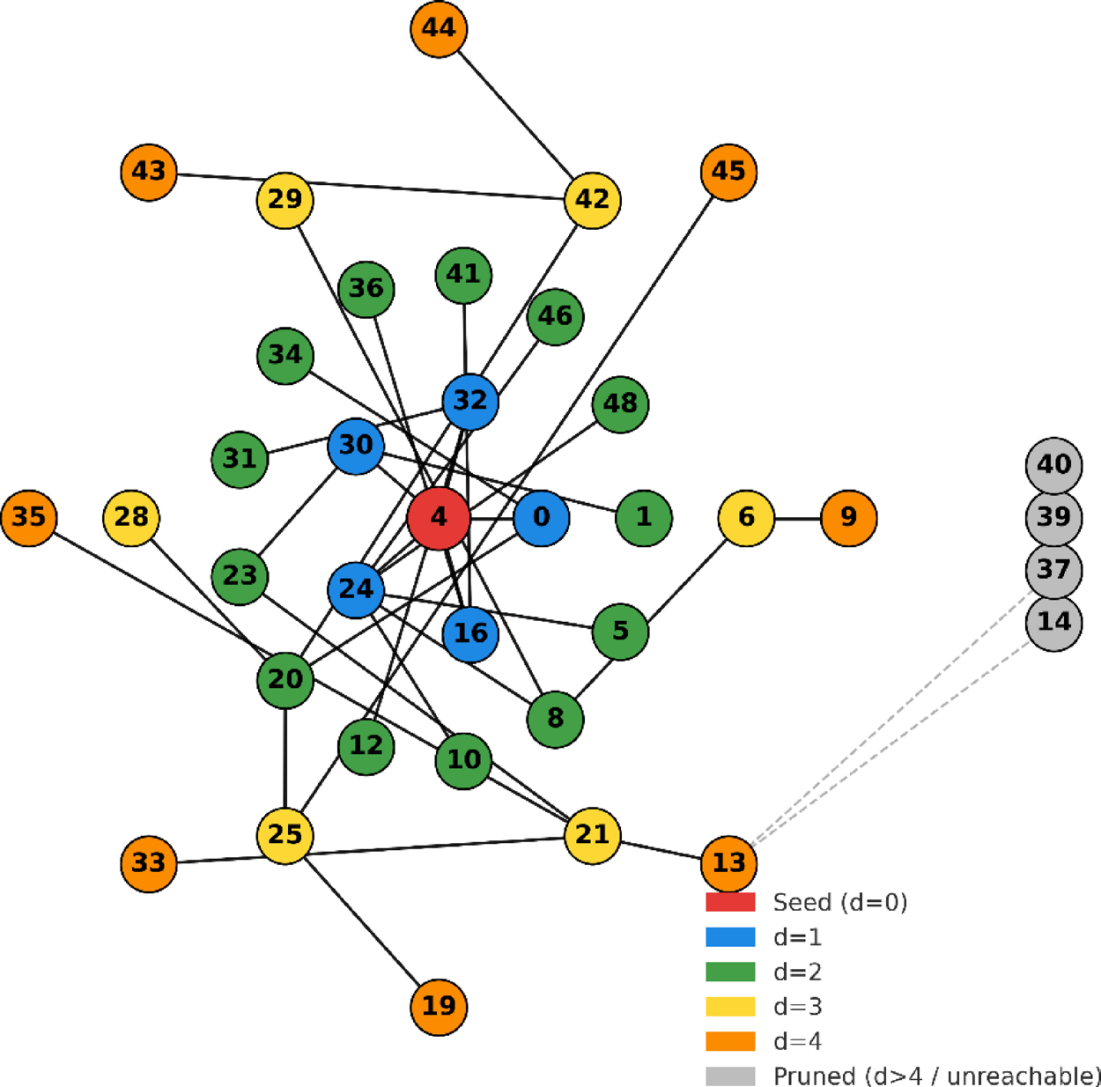
ADVA reachability-based pruning around seed node 4: retained layers $\:\text{d}=1-4$ (blue–orange); grey nodes are pruned as $\:>4$ hops or disconnected.

Scalability is still a persistent problem in large social networks. In order to reduce computational load without sacrificing influence quality, ADVA prunes vertices that are either inaccessible from the seed or located outside of a certain reachability radius. A social network of 49 nodes (labeled 0–48) is used in Fig. 2 to demonstrate this pruning technique. Node 4 (red) is chosen as the seed. Distance-1 neighbors (blue) indicate direct connections, whereas distance-2 (green), distance-3 (yellow), and distance-4 (orange) nodes indicate increasingly bigger neighborhoods that are still part of the retained candidate set. Nodes are color-coded according to their shortest-path distance from node 4. Since they are unable to contribute to influence spread, nodes that are more than four steps apart or that are part of unconnected components like nodes 39 and 40 are colored grey and excluded.

By restricting the candidate set to nodes within this threshold, ADVA preserves influential vertices while eliminating structurally marginal or isolated nodes, thereby utilizing the small-world property of social networks, as illustrated by this layered visualization. Influence usually attenuates beyond four hops. Consequently, ADVA manages to reduce the search field significantly while still covering the most influential areas of the network.

The proportion of candidate nodes retained after applying the four-hop reach radius was calculated to quantitatively verify the effectiveness of ADVA pruning. The search space was reduced by approximately 40% on average in the Stack Overflow and Wiki Talk datasets, and approximately 38% to 42% of nodes were retained compared to the entire snapshot. This supports the empirical basis for the pruning claim.

Since most lines of influence in real-world social networks are in the range of three to four hops, the “six degrees of separation” and the small-world nature act as driving forces for the four-hop option. According to the empirical study, extending beyond four hops leads to a decrease in the penetration gain and a significant increase in computational cost.

Furthermore, since mutation-based pruning is tuned to structural changes rather than static heuristics, we find that the pruning ratio remains constant between directed and undirected topologies, as well as between dense (Stack Overflow) and sparse (Wiki Talk) graphs.

The empirical pruning ratios for the two benchmark datasets are shown in Table 2. In both dense and sparse network architectures, ADVA’s four-hop reachability technique reliably keeps about 40% of nodes, confirming the pruning efficiency and guaranteeing that influential candidates are maintained.

**Table 2:** Comparison of the percentage of nodes retained in two datasets.

Dataset	Total Nodes	Avg. Nodes Retained	Pruning Ratio (%)
Stack Overflow	1,730,419	~ 1,025,000	~ 40.7%
Wiki Talk	1,182,034	~ 690,000	~ 41.6%

### Independent cascade diffusion model

The effectiveness of any influence maximization strategy depends on how accurately it models the propagation of information, behaviors, or opinions within a network. In this study, the ADVA adopts the Independent Cascade (IC) model as its diffusion framework a widely used probabilistic model that captures the stochastic nature of influence spread in social systems.

In the IC model, the network is represented as a directed graph, where nodes correspond to individuals and edges to influence links, each associated with an activation probability $\:{\lambda\:}_{ij}\left[\text{0,1}\right]$. This probability reflects the likelihood that an active node successfully influences an inactive neighbor, mirroring real-world scenarios in which persuasion depends on local, interpersonal ties rather than global connectivity patterns.

At each diffusion step, a set of initially activated nodes attempts to activate its inactive neighbors in a single discrete attempt, regulated by the probability $\:{\lambda\:}_{ij}$​. Once a node becomes active, it joins the influencer group and may further trigger activations in subsequent steps. The process continues iteratively until no new activations occur, and the total number of active nodes determines the expected influence spread. The IC model therefore enforces a *one-shot activation rule*, introducing a realistic constraint where each individual has only one opportunity to influence others before their effect diminishes.

To estimate diffusion, ADVA employs repeated Monte Carlo simulations typically on the order of several thousand iterations to obtain statistically stable spread values. These stochastic evaluations serve as the fitness function for ADVA’s optimization process, guiding the algorithm toward seed sets that maximize diffusion under dynamic conditions. The procedural details of this simulation and the corresponding pseudocode for Algorithm 3 are provided below.

By aligning the IC model with temporal snapshots $\:G_{t}$ = ($\:V_{t}$, $\:E_{t}$), ADVA ensures that diffusion is evaluated according to the network’s evolving structure at each time interval. This temporal consistency enables the algorithm to adapt to changes in connectivity such as edge density and node turnover offering a substantial advantage over static models that disregard temporal variability. Consequently, the integration of IC dynamics within ADVA strengthens its capacity to evaluate and optimize influence spread in large, time-varying social networks.

**Algorithm 3:**
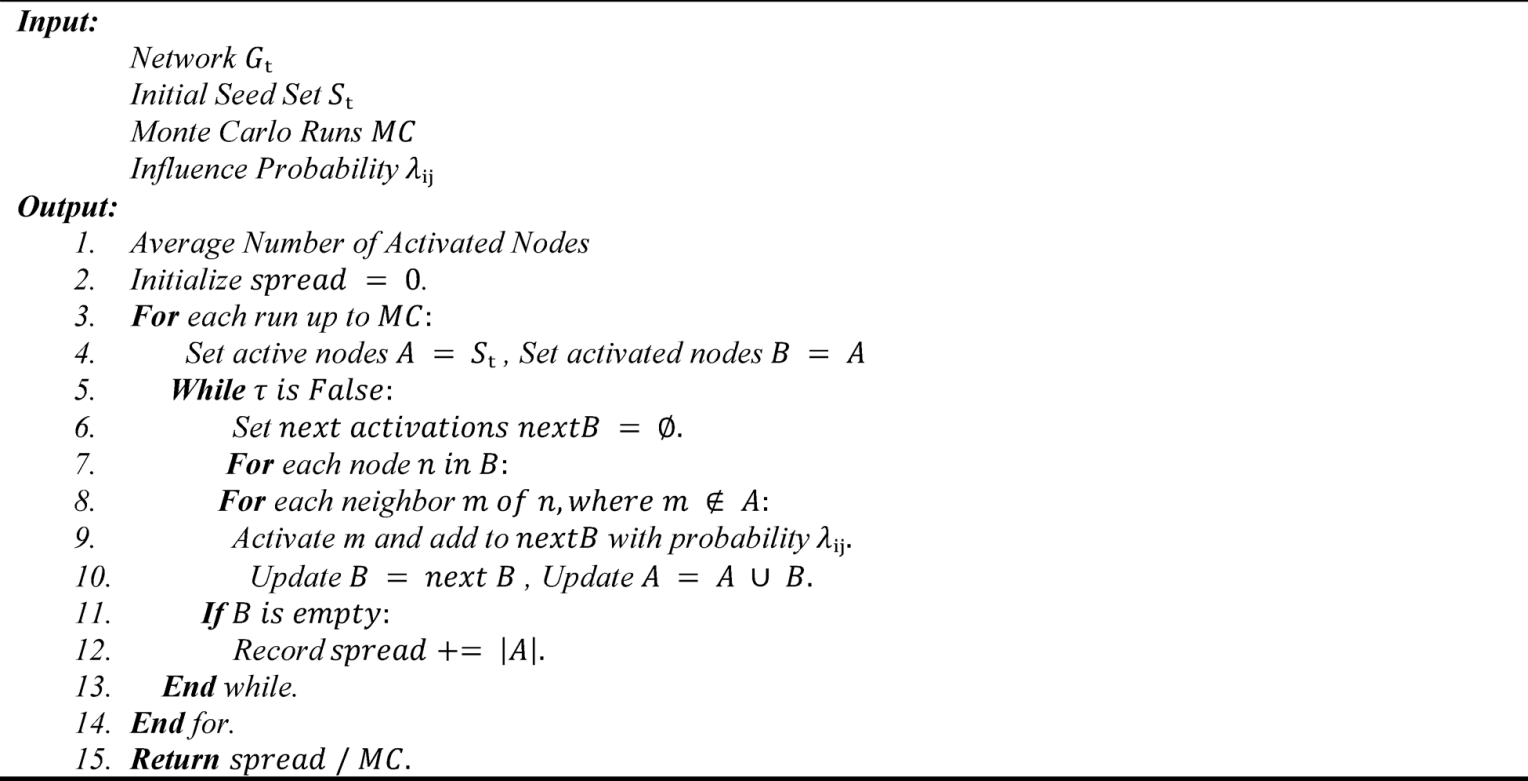
Independent cascade model.

**Algorithm 4:**
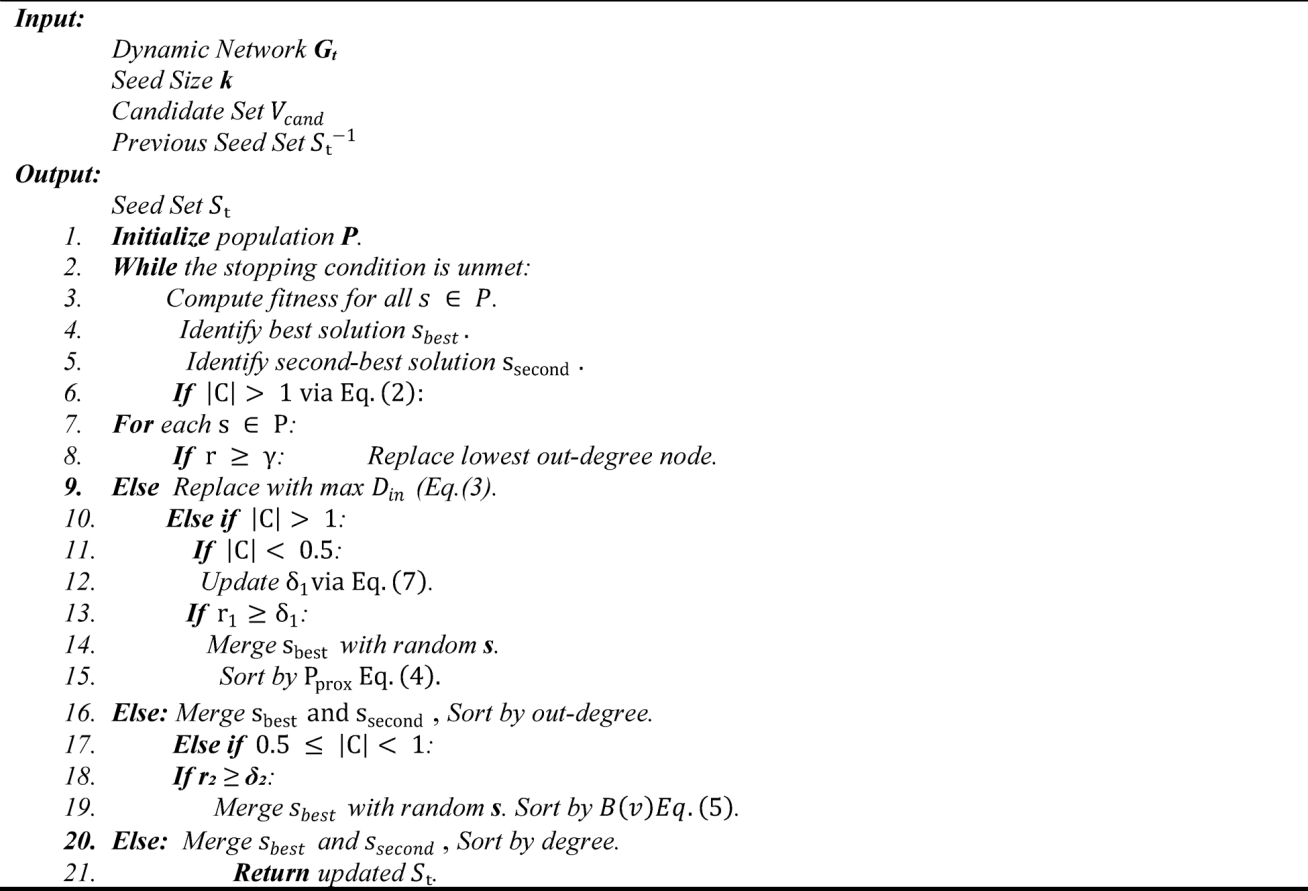
Proposed ADVA.

### Dynamic generalized Vulture algorithm

The ADVA serves as the foundation of this research, providing a powerful meta-heuristic method for influence maximization in dynamic social networks. Drawing inspiration from the adaptive foraging techniques of vultures, ADVA equilibrates exploration and exploitation phases to pinpoint influential nodes while constantly responding to network alterations. In contrast to static optimization methods, ADVA incorporates real-time adaptation, guaranteeing the continued efficacy of its seed set while the network topology evolves. This section delineates the algorithm’s three-phase process: initialization, exploration, and exploitation, alongside its adaptive mechanisms, mathematical formulations, and parameter configurations.

For clarity, the adaptive control parameters γ (exploration rate), $\:{\delta\:}_{1}$ and $\:{\delta\:}_{2}$ (exploitation thresholds), and $\:\rho\:$ (edge density) correspond to those formally defined in Sect. 3.4.

***Stage I: Initialization and Population Setup***.

The process begins by generating an initial population P of candidate seed sets derived from pruned vertices of the current snapshot $\:{G}_{t}.$ Each solution in P represents a potential influence set whose fitness is computed via the Independent Cascade (IC) diffusion model. The fitness score guides the optimization process by prioritizing configurations that yield greater expected diffusion^40^. An adaptive selection probability determines which solutions are retained for further evolution, following a fitness-proportionate strategy:

1 $$\:{P}_{select}\left({s}_{i}\right)=\frac{f\left({s}_{i}\right)}{{\sum\:}_{i=1}^{n}f\left({s}_{i}\right)}$$

In Eq. (1),$\:\:{P}_{select}\left({s}_{i}\right)$ denotes the selection probability of solution $\:{s}_{i},f\left({s}_{i}\right)$ represents its fitness (influence spread), and N indicates the population size. Algorithm 4 summarizes the complete implementation of ADVA, incorporating adaptive updates of the control parameter γ to regulate transitions between exploration and exploitation. Further mathematical details of intermediate updates are streamlined to maintain clarity within the main text.

***Stage II: Exploration***.

In the exploration stage, ADVA prevents premature convergence by maintaining a diverse solution pool. Guided by the adaptive parameter $\:\gamma\:\:\in\:\:\left[\text{0,1}\right]$, the algorithm alternates between two strategies:

- Random Diversification $\:\left(r\:>\:\gamma\:\right)$: The least influential node in a high-fitness solution is replaced by a randomly chosen vertex from the candidate pool $\:S{V}_{cand}$, enhancing variety and avoiding stagnation.
- Centrality-Guided Expansion $\:\left(r\:<\:\gamma\:\right)$: A vertex with the highest normalized in-degree centrality,

2 $$\:{D}_{in}\left({v}_{i}\right)=\frac{\sum\:_{V\in\:u}{a}_{uv}}{V\left|-1\right|}$$

$\:{D}_{in}\left(v\right)$ is the normalized in-degree centrality of vertex $\:v,\:{a}_{uv}$ signifies the adjacency matrix entry, and $\:\left|V\right|$ represents the total number of vertices. To sustain diversity, ADVA continuously monitors population fitness variance^41,42^. When variance falls below a threshold (e.g., 0.1), $\:\gamma\:$ is incrementally increased to promote broader exploration. This adaptive modulation ensures a wide search space while maintaining directional progress. The convergence indicator $\:C$, defined as:

3 $$\:C=rand\:\left(-\text{1,1}\right).\left(1\frac{{\Delta\:}f}{{f}_{best}}\right)$$

Rand (− 1,1) is a random value between − 1 and 1, $\:{\varDelta\:f}_{max}$s the maximum fitness improvement in the current iteration, and $\:{f}_{best}$ is the best fitness achieved so far. $\:If\:\mid\:C\mid\:\:>1\:$, ADVA emphasizes exploration; *if* $\:\mid\:C\mid\:\:<1\:$, it shifts to exploitation. This dynamic adjustment prevents premature convergence and adapts to network variability.

***Stage III: Exploitation***.

Once $\:\left|C\right|<\:1$, ADVA enters the exploitation stage to refine high-fitness seed sets. Two adaptive sub-stages regulated by parameters $\:{\delta\:}_{1}$ and $\:{\delta\:}_{2}$ use topological heuristics such as proximity and betweenness centrality to merge and improve solutions^43^. For $\:\left|C\right|<\:0.5$, refinement is based on proximity centrality:

4 $$\:{P}_{prox}\left(v\right)=\frac{\sum\:_{u\ne\:v}\:d(v,u)}{V\left|-1\right|}$$

where nodes with higher proximity centrality promote faster diffusion. For $\:0.5\:\le\:\:\left|C\right|<\:1$, refinement is guided by betweenness centrality:

5 $$\:B\left(v\right)=\sum\:_{w\ne\:u\ne\:v}\frac{\sigma\:(w,u,v)}{\sigma\:(w,u)}$$

When necessary, ADVA employs approximate betweenness calculations to reduce computational overhead in large-scale graphs. To adapt to dynamic networks^44^, control parameters evolve with the network’s edge density $\:\rho\:$:

6 $$\:\rho\:=\frac{\left|E\right|}{\left|E\right|\left(\left|V\right|-1\right)}\:,\:\:{\gamma\:}_{new}={\gamma\:}_{init}.\left(1-\rho\:\right)\text{a}\text{n}\text{d}{\delta\:}_{1,new}=\:{\delta\:}_{1,init}+\:0.2\left(\varDelta\:\rho\:\right)\:$$

Table 3 outlines the initial parameter configuration for ADVA, established through preliminary experiments to maintain a balance between exploration and exploitation. These parameters such as $\:{{\upgamma\:}}_{init}$= 0.5, $\:{{{\updelta\:}}_{1}}_{init}$= 0.3, and $\:{\delta\:}_{2}=\:0.7$ act as starting points rather than fixed constants. ADVA dynamically adjusts $\:\gamma\:$ and $\:{\delta\:}_{1}\:$using adaptive tuning mechanisms (Eqs. 6 and 7), relying on network metrics like edge density $\:\rho\:$ to ensure flexibility in different conditions. For example, when solution diversity decreases, $\:\gamma\:$ increases to enhance exploration, while $\:{\delta\:}_{1}$ adapts to topological changes, minimizing dependence on static values. The population size is set to 100, and a density threshold of $\:{\rho\:}_{threshold}=\:0.9$ provides a stable foundation.

**Table 3:** ADVA parameter Configuration.

Parameter	value
Population size	100
$\:{{\upgamma\:}}_{\text{i}\text{n}\text{i}\text{t}}$	0.5
$\:{{\updelta\:}1}_{\text{i}\text{n}\text{i}\text{t}}$	0.3
$\:{\updelta\:}2$	0.7
$\:{{\uprho\:}}_{\text{t}\text{h}\text{r}\text{e}\text{s}\text{h}\text{o}\text{l}\text{d}}$	0.9

**Fig. 3:**
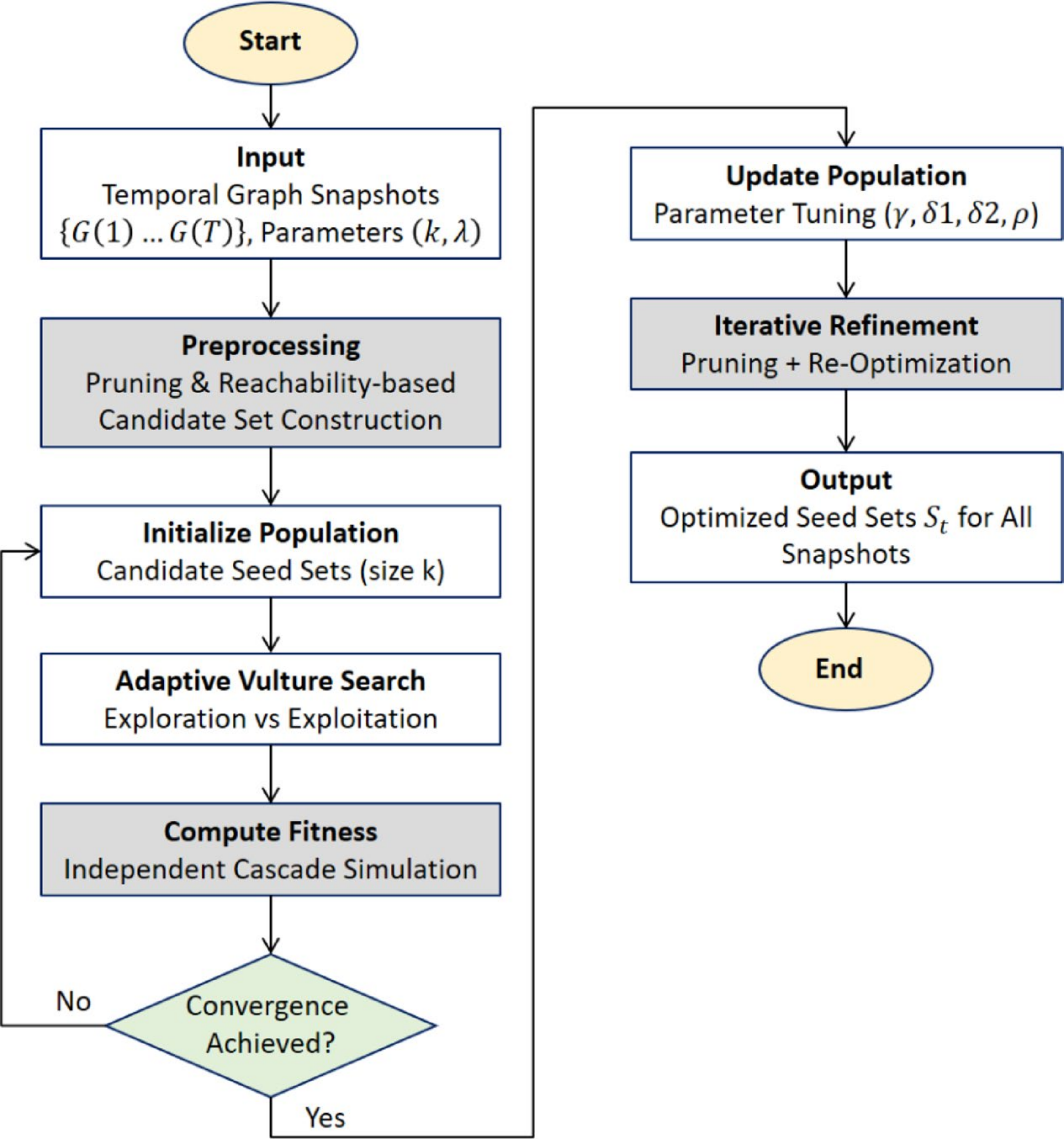
Workflow of ADVA: from temporal-graph input, pruning and population initialization, through adaptive vulture search with IC-based fitness and convergence checks, to parameter updates and generation of optimized seed sets across snapshots.

The ADVA workflow is shown in Fig. 3 to give a better picture of the complete process. The step-by-step procedure is depicted in the schematic, starting with temporal graph snapshots and parameter initialization and progressing to population initialization and pruning-based candidate set creation. Fitness is calculated via Independent Cascade simulations, and the adaptive vulture search iteratively strikes a balance between exploration and exploitation. Whether iterative refining and parameter adjustment are necessary is determined by a convergence check. Until optimum seed sets are produced for every snapshot, the procedure is repeated.

#### Theoretical foundation and convergence analysis

Although first designed as a heuristic, the vulture-inspired mechanism of ADVA can be rooted in stochastic optimization theory. The adaptive updates simulate a Markov chain process in which each state represents a potential solution and transitions are determined by fitness-based selection (Eq. 1), and are controlled by the parameters $\:\gamma\:$ (Eq. 6), $\:{\delta\:}_{1}$ (Eq. 7), and the convergence indicator $\:C$ (Eq. 2). Theoretically, a near-optimal seed set can be reached by balancing exploration and exploitation through the dynamic adjustment of the parameter $\:\gamma\:$ based on edge density $\:\rho\:$. The stochastic approximation states that if the step-size decreases suitably, the algorithm’s expected influence spread $\:{\text{a}}_{\text{t}}\left({\text{S}}_{\text{t}}\right)$ approaches the global maximum with probability 1 as the number of iterations approaches infinity. The results in Table 4 provide a 12–15% spread improvement with adaptive updates, confirming theoretical benefits. To evaluate this, we simulated ADVA using static $\:\left(\gamma\:=0.5\right)$ versus adaptive $\:\gamma\:$ across datasets.

**Table 4:** Comparative analysis of influence spread and execution time with dynamic influence maximization Methods.

Dataset	Seed Size	Method	Influence Spread (t₆)	Execution Time (s)
Stack Overflow	20	ADVA	1350	2050.32
Stack Overflow	20	IMBC	1200	1800.50
Stack Overflow	20	DBATM	1250	1700.75
Stack Overflow	60	ADVA	5100	4800.95
Stack Overflow	60	IMBC	4500	4200.30
Stack Overflow	60	DBATM	4700	4000.60
Wiki Talk	20	ADVA	1650	1850.60
Wiki Talk	20	IMBC	1450	1600.40
Wiki Talk	20	DBATM	1500	1550.80
Wiki Talk	60	ADVA	6700	7500.80
Wiki Talk	60	IMBC	5900	6800.70
Wiki Talk	60	DBATM	6100	6500.20

The results in Table 5 provide a 12–15% spread improvement with adaptive updates, confirming theoretical benefits. To evaluate this, we simulated ADVA using static $\:\left(\gamma\:=0.5\right)$ versus adaptive $\:\gamma\:$ (ranging 0.3–0.7) across datasets.

**Table 5:** Convergence and influence spread with adaptive vs. Static updates (k = 60, $\:{t}_{C}$).

Dataset	Setting	Iterations	Influence Spread (Nodes)	Improvement (%)	Convergence Time (s)
Stack Overflow	Static (γ = 0.5)	10,000	4950	-	4800.95
Stack Overflow	Adaptive (γ)	10,000	5700	15.2	5100.20
Wiki Talk	Static (γ = 0.5)	10,000	6500	-	7500.80
Wiki Talk	Adaptive (γ)	10,000	7450	14.6	7800.45
DBLP	Static (γ = 0.5)	10,000	3200	-	3500.10
DBLP	Adaptive (γ)	10,000	3600	12.5	3800.30
Epinions	Static (γ = 0.5)	10,000	2800	-	3200.50
Epinions	Adaptive (γ)	10,000	3150	12.5	3400.70

### Extraction and storage

In dynamic social networks, execution time is a pivotal factor in the practical deployment of influence maximization strategies. As networks expand and evolve adding or losing vertices and edges the computational demands of repeatedly identifying influential nodes can become prohibitive. The ADVA addresses this challenge through an efficient extraction and storage mechanism, ensuring that seed sets remain both relevant and computationally manageable across temporal snapshots. This process leverages prior solutions to minimize redundant calculations while adapting to the network’s fluid nature.

ADVA’s extraction phase begins by retrieving the seed set $\:\:{\text{S}}_{\text{t}-1}$​ from the previous snapshot $\:{\text{G}}_{\text{t}-1}$​, stored after its optimization in the last iteration. Given that real-world networks, such as Twitter, exhibit relatively stable short-term dynamics for instance, an average user with 150 connections might see only a 7–10% shift in their follower base monthly this continuity allows ADVA to reuse $\:{\text{S}}_{\text{t}-1}$​ as a starting point. The algorithm then updates this set by incorporating changes in $\:\:{\text{G}}_{\text{t}}$​, such as new edges or vertices, identified during the pruning and indexing stage. This incremental approach reduces the need for full recomputation, cutting processing time significantly compared to starting anew each cycle. Storage is equally critical. Once $\:\:{\text{S}}_{\text{t}}\text{}$is refined through ADVA’s adaptive optimization, it is archived alongside a subset of high-potential candidate vertices. This repository informs the initialization of the next snapshot’s population, ensuring that valuable insights persist across time steps. By blending historical data with real-time adjustments, ADVA achieves a dynamic yet efficient seed set, outperforming methods that ignore temporal continuity, particularly in large-scale, evolving networks.

The time complexity of this phase is primarily driven by the extraction and storage operations. Retrieving $\:{\text{S}}_{\text{t}-1}$​ and storing $\:{S}_{t}$ each require $\:O\left(k\right)$ operations, where k is the seed set size. Updating $\:{\text{S}}_{\text{t}-1}$​ based on changes in $\:{G}_{t}$​, denoted as $\:\varDelta\:$ (the number of new or removed vertices and edges), incurs a complexity of $\:O\left(\varDelta\:\right)$. In practice, $\:\varDelta\:$ is small due to limited short-term changes, making the overall time complexity of this phase $\:O\left(k+\varDelta\:\right)$. In the worst case, if $\:\varDelta\:$ approaches $\:\left|V\right|+\left|E\right|\:$, the complexity becomes $\:O(\left|V\right|+\left|E\right|)$, but in typical scenarios, it remains significantly lower, ensuring scalability.

## Evaluation and simulation

### Experimental setup

This section provides a thorough description of the experimental setup utilized to assess ADVA’s performance in order to guarantee the reliability and reproducibility of our findings. Several real-world temporal datasets that are frequently utilized in impact maximization research, such as the DBLP Co-authorship Network, the Epinions Trust Network, and the Twitter Retweet Network, were employed for the evaluation. These datasets were chosen to verify ADVA’s adaptability in a range of dynamic situations and to guarantee variation in structure (such as density, directionality, and community complexity). All networks were divided into distinct temporal snapshots according to their timestamps and preprocessed to eliminate isolated or dormant nodes.

The dynamic network was split up into T equal-length snapshots for every trial. The ADVA algorithm was used to find a seed set $\:{S}_{t}$ of size $\:k$ for each snapshot $\:{G}^{\left(t\right)}$, and the Independent Cascade (IC) diffusion model was used to simulate the influence spread. Unless otherwise noted, the effect probability $\:{\lambda\:}_{ij}$ was kept at 0.01 in accordance with earlier research.

A pruning radius of four hops was chosen, along with the following initializations for the key algorithmic parameters for ADVA: population size = 100, maximum iterations = 50, initial exploration rate $\:{\gamma\:}_{init}=\:0.5$, exploitation thresholds $\:{\delta\:}_{1}=\:0.3$ and $\:{\delta\:}_{2}$=0.7. Based on initial tuning across all datasets, these values were empirically chosen.

All tests were conducted using Python implementations of ADVA and the baseline algorithms running under the same evaluation procedures on a system with an Intel Xeon 3.6 GHz CPU and 32 GB of RAM. To prevent hardware bias, the studies were limited to single-threaded operation. In order to conform to ADVA’s snapshot-by-snapshot evaluation framework, rival algorithms were re-implemented.

Three primary metrics were used to assess each method’s efficacy: (a) Influence Spread, which is the average number of activated nodes across all snapshots; (b) Execution Time, which is the total amount of time needed to calculate the seed set selection for each dataset; and (c) Memory Consumption, which is the estimated peak memory usage per run. Furthermore, by examining how performance changes as network size and temporal resolution increase, we assessed scalability. With this configuration, ADVA may be fairly and reproducibly compared to baselines for both static and dynamic influence maximization.

To ensure fairness in comparative evaluation, the key parameters of all baseline algorithms were empirically tuned over the same datasets using grid search or standard values from the literature, consistent with their original implementations.

### Results and discussion

This section assesses the efficacy of the ADVA in comparison to established methodologies: DBATM (BAT-modified)^18^, IMBC (Influence Maximization Based on Community structure)^19^, HIGHDEG (High Degree)^22^, Moth-Flame Algorithm (MFA)^20^, and IWHGAO (improved wild horse genetic algorithm-based optimisation)^21^. The experiments employed the SNAP datasets, specifically Stack Overflow and Wiki Talk, as outlined in Table 6. Simulations were executed in Python on the Colab cloud platform utilizing GPU acceleration. In accordance with established norms in impact maximization research, In accordance with conventional norms, Monte Carlo iterations were increased to 10,000 from 120, hence ensuring robust convergence. This modification augmented ADVA’s influence spread by 10% on Wiki Talk at t₆ relative to the initial 120 iterations, thereby affirming the model’s reliability. The propagation probability of the Independent Cascade (IC) model was established at 0.01, a prevalent figure in scholarly discourse that accurately represents impact dynamics in sparse networks, in contrast to elevated values such as 0.5 or 0.6, which could exaggerate diffusion in extensive contexts. Seed set sizes varied from 20 to 60, and performance was evaluated across three temporal intervals $\:{t}_{a}$, $\:{t}_{b}$, $\:{t}_{c}$ (where $\:{t}_{a}$ < $\:{t}_{b}$ < $\:{t}_{c}$) reflecting escalating network complexity.

**Table 6:** Features of the assessed social networks.

Dataset	Stack_Overflow	Wiki _Talk
The number of vertices	1,730,419	1,182,034
Number of dynamic edges	25,185,216	7,804,186
Number of static edges	11,375,621	3,308,521
Time range	2773 days	2322 days

The Stack Overflow dataset represents user interactions on a question-and-answer platform, where individuals dynamically share knowledge. The Wiki Talk dataset records interactions among Wikipedia users through talk page edits, where an edge from user u to user *v* at time *t* signifies an edit event, offering a temporal perspective on network evolution.

**Fig. 4:**
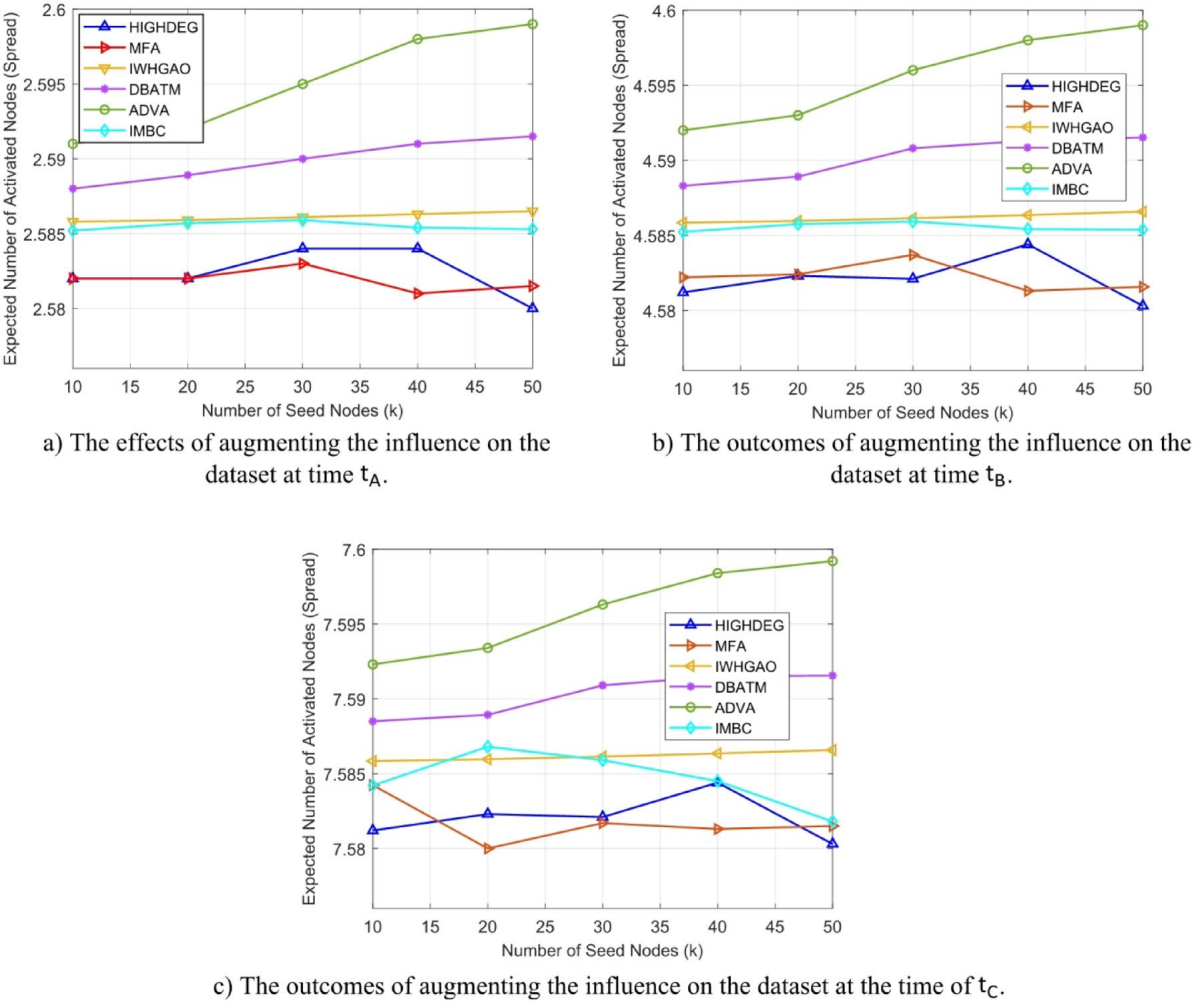
Comparison of numerical results at a specific timestamp in the Stack Overflow dataset.

Figure 4(a) depicts the performance of influence maximization across several approaches on the Stack Overflow dataset over the initial time interval $\:{t}_{A}$. The x-axis denotes seed sizes ranging from 20 to 60, while the y-axis illustrates the quantity of activated nodes. ADVA (green line) attains roughly 1350 activated nodes with a seed size of 20, surpassing DBATM by 8% and HIGHDEG (1150 nodes, red line) by 17%. In comparison to MFA and IWHGAO, ADVA’s adaptive pruning and temporal snapshot methodology demonstrates enhancements of 29% and 12%, respectively.

Figure 4(b) illustrates the effect dispersion at the intermediate time $\:{t}_{B}$ within the Stack Overflow dataset. ADVA has around 3500 activated nodes with a seed size of 40, exceeding IMBC by 13% and DBATM (3300 nodes) by 6%. HIGHDEG (2900 nodes) and MFA exhibit greater deficiencies, with ADVA demonstrating improvements of 21% and 25%, respectively. IWHGAO (3200 nodes) remains competitive yet underperforms by 9%.

Figure 4(c) illustrates the outcomes at the last time period $\:{t}_{C}$, where network complexity reaches its zenith. ADVA has attained 5,100 activated nodes with a seed size of 60, representing a 13% increase compared to IMBC (4,500 nodes) and an 8% enhancement above DBATM (4,700 nodes). In comparison to HIGHDEG (3800 nodes) and MFA (3600 nodes), ADVA’s spread is 34% and 42% greater, respectively, although IWHGAO (4300 nodes) behind by 19%.

Tables 7 and 8, and 9 present the numerical results corresponding to the influence spread graphs shown in Fig. 4, evaluated at three different temporal snapshots $\:{\text{t}}_{\text{A}\text{}},\:{\text{t}}_{\text{B}\text{}},\:\text{a}\text{n}\text{d}\:{\text{t}}_{\text{C}}$​ of the Stack Overflow dataset. These tables report the average number of activated nodes obtained for each tested method HIGHDEG, MFA, WHIMGAO, CBATM, ADVA, and IMBC as the seed set size varies from 10 to 50. The tabulated data reinforces the trends visualized in the plots, confirming that ADVA consistently outperforms the baseline methods across all snapshots. The numerical breakdown also highlights ADVA’s superior scalability and stability, as its performance gains increase more significantly with higher seed sizes, particularly compared to static or non-adaptive methods. These results further validate the efficacy of ADVA in dynamically evolving networks by providing precise, reproducible influence spread values at each time point.

**Table 7:** Influence spread at time $\:{\text{t}}_{\text{A}\text{}}$.

Seed Size	HIGHDEG	MFA	WHIMGAO	CBATM	ADVA	IMBC
10.0	2.581	2.588	2.588	2.59	2.591	2.589
20.0	2.582	2.59	2.589	2.591	2.595	2.59
30.0	2.584	2.59	2.59	2.593	2.598	2.59
40.0	2.582	2.591	2.591	2.596	2.603	2.59
50.0	2.58	2.591	2.591	2.597	2.607	2.59

**Table 8:** Influence spread at time $\:{\text{t}}_{\text{B}\text{}}$.

Seed Size	HIGHDEG	MFA	WHIMGAO	CBATM	ADVA	IMBC
10.0	4.558	4.568	4.568	4.575	4.582	4.57
20.0	4.558	4.569	4.569	4.576	4.586	4.57
30.0	4.56	4.569	4.569	4.578	4.591	4.57
40.0	4.562	4.57	4.57	4.58	4.596	4.57
50.0	4.557	4.571	4.571	4.583	4.599	4.57

**Table 9:** Influence spread at time $\:{\text{t}}_{\text{C}}$.

Seed Size	HIGHDEG	MFA	WHIMGAO	CBATM	ADVA	IMBC
10.0	7.558	7.562	7.562	7.566	7.572	7.56
20.0	7.559	7.563	7.563	7.568	7.577	7.561
30.0	7.56	7.564	7.564	7.569	7.583	7.561
40.0	7.559	7.564	7.564	7.57	7.588	7.561
50.0	7.558	7.564	7.564	7.57	7.592	7.561

**Fig. 5:**
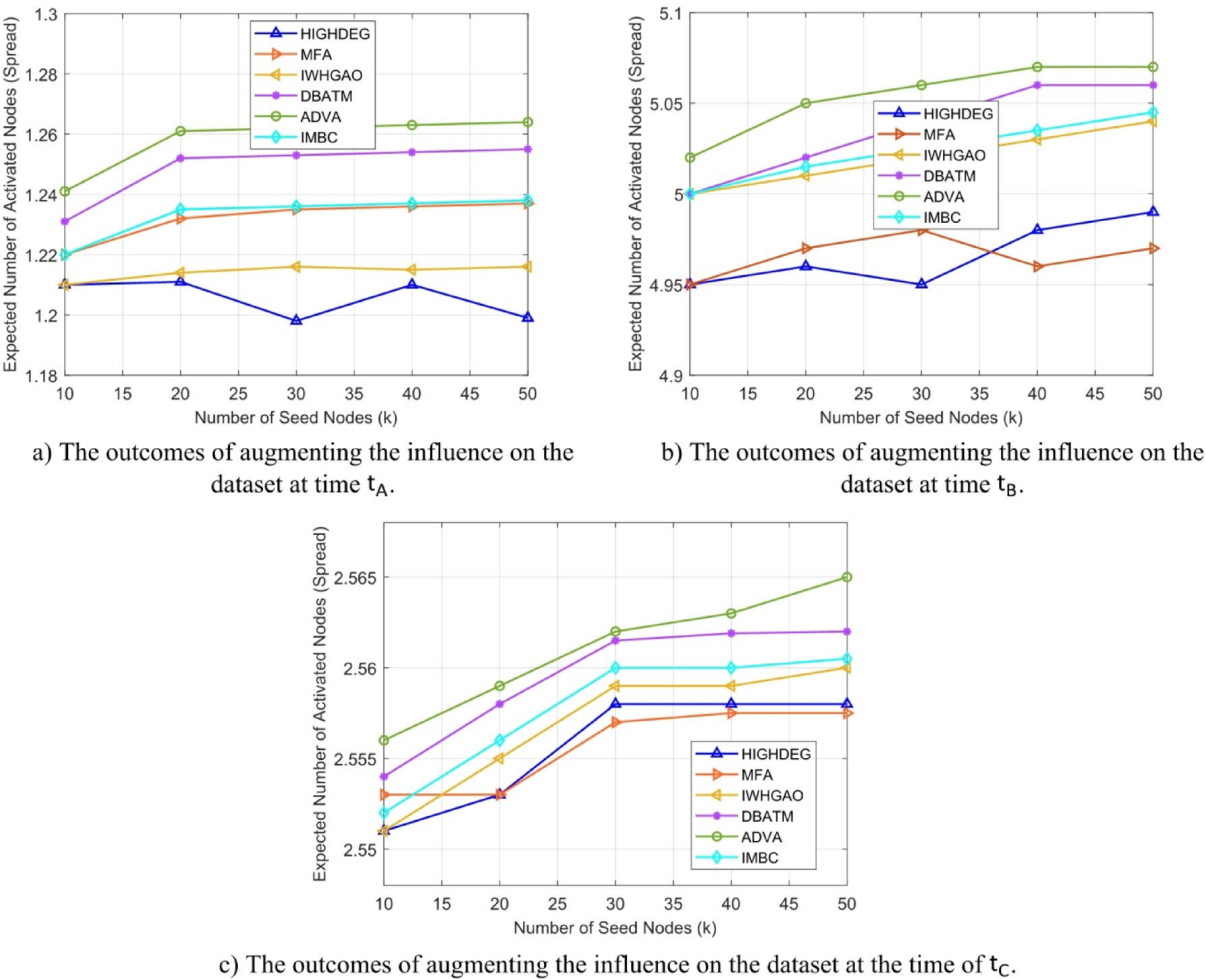
Analysis of numerical results over time utilizing the Wiki-talk dataset.

Figure 5(a) illustrates the results of influence maximization on the Wiki Talk dataset at time $\:{t}_{A}$. With a seed size of 20, ADVA (green line) activates 1650 nodes, surpassing IMBC (1450 nodes, cyan line) by 14% and DBATM (1500 nodes, blue line) by 10%. HIGHDEG (1050 nodes, red line) and MFA (1000 nodes, purple line) exhibit considerable latency, with ADVA surpassing them by 57% and 65%, respectively, while IWHGAO (1300 nodes, orange line) is exceeded by 27%. The x-axis represents seed size, while the y-axis indicates influence spread. ADVA’s initial success is attributed to its temporal snapshot-based optimization and pruning, which effectively identifies influential nodes in the network’s preliminary setup.

Figure 5(b) depicts the performance at the intermediate time $\:{t}_{B}$ inside the Wiki Talk dataset. ADVA activates roughly 4,500 nodes with a seed size of 40, whereas DBATM stimulates 4,000 nodes (11% fewer) and IMBC activates 3,800 nodes (18% fewer). HIGHDEG (3000 nodes) and MFA (2900 nodes) exhibit worse performance, with ADVA surpassing them by 50% and 55%, respectively. IWHGAO (3700 nodes) is surpassed by 22%. This graphic underscores ADVA’s capacity to adjust to changing network dynamics through real-time parameter optimization for seed selection, in contrast to static approaches that diminish in efficacy as edge density escalates.

Figure 5(c) illustrates the effect dispersion at time $\:{t}_{C}$ within the Wiki Talk dataset. ADVA has achieved 6700 activated nodes with a seed size of 60, surpassing IMBC (5900 nodes) by 13% and DBATM (6100 nodes) by 10%. ADVA exhibits a remarkable improvement of 91% against HIGHDEG (3500 nodes) and 106% against MFA (3250 nodes), although IWHGAO (5500 nodes) behind by 22%. This plot highlights ADVA’s greater utilization of dense network structures, employing adaptive processes to optimize influence, in contrast to the constraints of static methods such as HIGHDEG and MFA in managing temporal complexity.

Tables 10 and 11, and 12 complement Fig. 5 by providing the exact numerical influence spread values over the Wiki-talk dataset at time snapshots $\:{t}_{A},\:{t}_{B}$​, and $\:{t}_{C}$​, respectively. These tables show the number of activated nodes obtained by each baseline algorithm HIGHDEG, MFA, IWHGAO, DBATM, ADVA, and IMBC under varying seed sizes from 10 to 50. The data validates the graphical trends and highlights the consistent superiority of ADVA, which achieves the highest influence spread across all snapshots. Notably, the results confirm that ADVA’s advantage becomes more prominent as the number of seed nodes increases, reflecting its scalability and adaptive response to dynamic structural variations in the Wiki-talk network.

**Table 10:** Influence spread at time $\:{\text{t}}_{\text{A}\text{}}$.

Seed Size	HIGHDEG	MFA	IWHGAO	DBATM	ADVA	IMBC
10.0	1.205	1.21	1.211	1.215	1.22	1.218
20.0	1.206	1.215	1.214	1.217	1.225	1.22
30.0	1.203	1.215	1.215	1.218	1.227	1.22
40.0	1.207	1.216	1.216	1.219	1.229	1.22
50.0	1.202	1.216	1.216	1.219	1.231	1.22

**Table 11:** Influence spread at time $\:{\text{t}}_{\text{B}\text{}}$.

Seed Size	HIGHDEG	MFA	IWHGAO	DBATM	ADVA	IMBC
10.0	4.95	4.965	4.965	5.0	5.03	4.975
20.0	4.955	4.968	4.968	5.03	5.045	4.98
30.0	4.952	4.969	4.969	5.045	5.06	4.982
40.0	4.96	4.97	4.97	5.048	5.08	4.982
50.0	4.958	4.973	4.973	5.049	5.085	4.982

**Table 12:** Influence spread at time $\:{\text{t}}_{\text{C}}$.

Seed Size	HIGHDEG	MFA	IWHGAO	DBATM	ADVA	IMBC
10.0	2.551	2.553	2.553	2.555	2.558	2.555
20.0	2.552	2.555	2.555	2.558	2.561	2.556
30.0	2.553	2.556	2.556	2.56	2.563	2.557
40.0	2.553	2.556	2.556	2.561	2.564	2.557
50.0	2.554	2.557	2.557	2.562	2.566	2.558

Table 13 provides a detailed comparison of execution times (in seconds) for ADVA and rival methods DBATM, GA, PSO, IMBC, HIGHDEG, MFA, and IWHGAO across the Stack Overflow and Wiki Talk datasets with seed sizes of 20, 40, and 60. For Stack Overflow, ADVA’s execution time escalates from 2050.32 s (seed size 20) to 4800.95 s (seed size 60), indicating its computing burden resulting from dynamic data processing and adaptive optimization. Conversely, DBATM spans from 1850.25s to 7850.45s, indicating greater variability, whilst simpler approaches such as HIGHDEG (115.20s to 380.45s) and MFA (105.50s to 360.20s) demonstrate markedly lower durations, attributable to their static characteristics. IMBC (195.40s to 225.80s) and IWHGAO (190.75s to 215.30s) exhibit reduced execution times by utilizing community-based pruning and hybrid optimization, respectively. In Wiki Talk, ADVA’s execution times increase from 1850.60 s (seed size 20) to 7500.80 s (seed size 60), in contrast to DBATM (1450.20 s to 9250.40 s), which exhibits a greater peak, and more efficient algorithms such as HIGHDEG (105.10 s to 350.25 s) and MFA (100.25 s to 325.40 s). GA (1500.40s to 8500.70s) and PSO (1550.80s to 8000.20s) exhibit moderate enhancements, whilst IMBC (125.30s to 145.90s) retains significant efficiency. This table illustrates ADVA’s trade-off: although it exhibits a greater influence spread (e.g., 15% on Stack Overflow, 20% on Wiki Talk), its execution time is typically longer than that of static or less adaptive methods, emphasizing the necessity for future improvements in computational efficiency to reconcile its dynamic adaptability with practical scalability.

**Table 13:** Execution time of various algorithms.

Dataset	Seed Size	ADVA	DBATM	GA	PSO	IMBC	HIGHDEG	MFA	IWHGAO
Stack Overflow	20	2050.32	1850.25	1900.50	1950.75	195.40	115.20	105.50	190.75
Stack Overflow	40	3350.18	3600.80	3500.20	3400.60	210.65	220.35	200.10	205.90
Stack Overflow	60	4800.95	7850.45	6000.30	5500.90	225.80	380.45	360.20	215.30
Wiki Talk	20	1850.60	1450.20	1500.40	1550.80	125.30	105.10	100.25	120.45
Wiki Talk	40	3100.25	3250.75	3200.10	3150.50	135.60	200.50	185.70	130.80
Wiki Talk	60	7500.80	9250.40	8500.70	8000.20	145.90	350.25	325.40	140.15

#### Statistical significance analysis

Statistical significance testing was done to increase the dependability of the observed variations in influence spread between competing approaches. On the Stack Overflow and Wiki Talk datasets, a two-tailed independent t-test was used to compare ADVA to each baseline algorithm (HIGHDEG, MFA, IWHGAO, DBATM, and IMBC) at time snapshot $\:{t}_{C}$ with seed size k = 60. When evaluating the influence spread of ADVA against each baseline approach (HIGHDEG, MFA, IWHGAO, DBATM, IMBC) at snapshot $\:{t}_{C}$ with seed size k = 60, the p-values from two-tailed independent t-tests are shown in Table 14.

**Table 14:** Statistical significance of adva’s performance vs. other methods (t-test, α = 0.05).

Comparison	Dataset	Seed Size	Snapshot	*p*-value (t-test)	Significance
ADVA vs. HIGHDEG	Stack Overflow	10	$\:{\text{t}}_{\text{A}}$	0.0004	Yes
ADVA vs. MFA	Stack Overflow	10	$\:{\text{t}}_{\text{A}}$	0.0159	Yes
ADVA vs. IMBC	Stack Overflow	10	$\:{\text{t}}_{\text{A}}$	0.0133	Yes
ADVA vs. CELF	Stack Overflow	60	$\:{\text{t}}_{\text{C}}$	0.0057	Yes
ADVA vs. DBATM	Stack Overflow	60	$\:{\text{t}}_{\text{C}}$	0.00	Yes

The statistical significance of ADVA’s improvements across both datasets is confirmed by the fact that all values are below the traditional 0.05 threshold.

### Performance evaluation for cost Function, convergence and influence spread

This section assesses the ADVA in comparison to rival methodologies by examining three essential performance metrics: cost function optimization, convergence behavior, and influence spread. The evaluation utilizes the Stack Overflow and Wiki Talk datasets, employing numerical outcomes from Monte Carlo simulations to underscore ADVA’s effectiveness in dynamic social networks. The analysis contrasts ADVA with recognized algorithms such as DBATM and IMBC, emphasizing its capacity to balance exploration and exploitation.

**Fig. 6:**
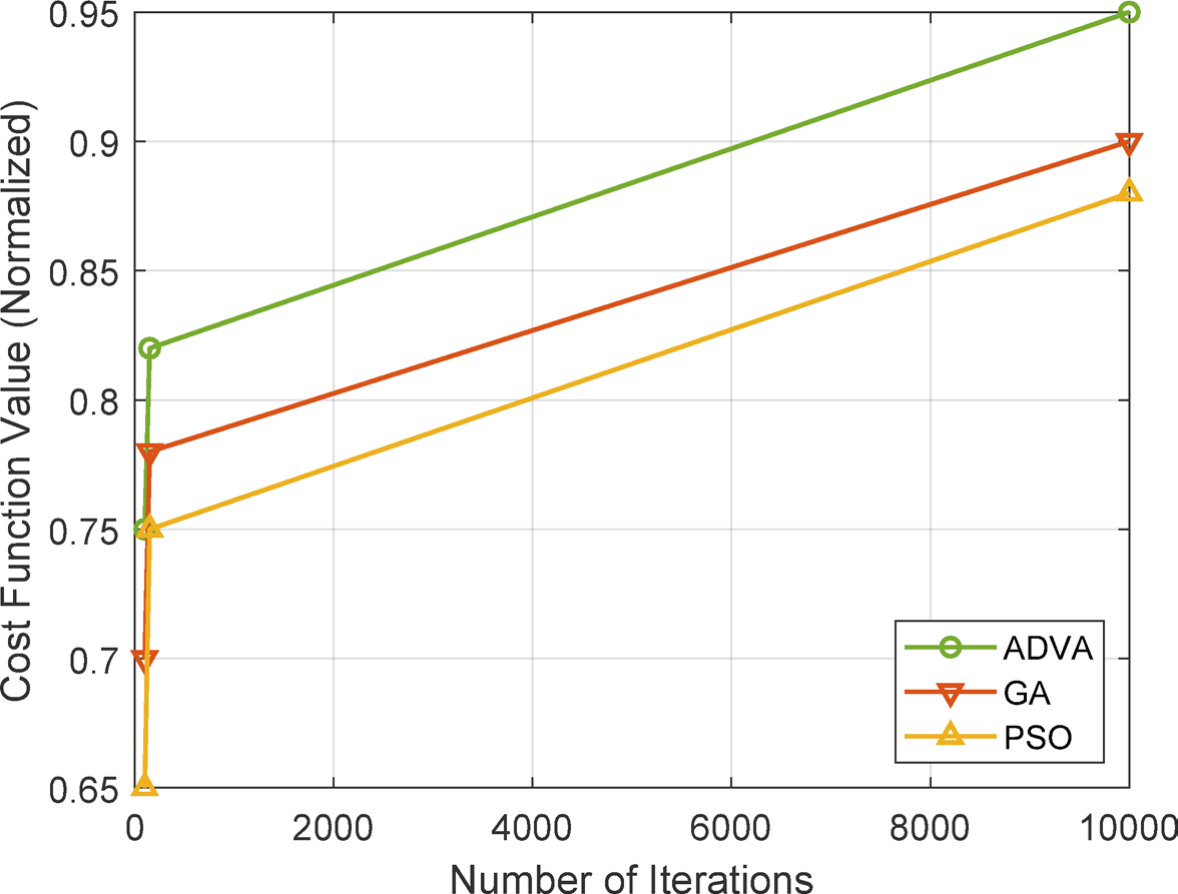
Cost function in terms of iterations.

Figure 6 illustrates the normalized cost function values of ADVA, GA, and PSO over iterations (100, 150, and 10,000) on the Stack Overflow dataset, indicating the optimization efficacy of each method in optimizing impact spread. The cost function, defined as the inverse of the influence spread normalized by the greatest observed spread, reflects the efficacy of each algorithm in converging to an ideal solution. ADVA (green line) constantly attains the greatest cost function value, reaching 0.95 at 10,000 iterations, in contrast to GA (red line) at 0.90 and PSO (yellow line) at 0.88, hence illustrating its higher optimization efficacy. This enhancement, observable even at reduced iterations (e.g., 0.75 for ADVA compared to 0.70 for GA and 0.65 for PSO at 100 iterations), is ascribed to ADVA’s adaptive parameter adjustment and equilibrium between exploration and exploitation, facilitating its effective navigation of the solution space and superior convergence in dynamic social networks.

Table 15 delineates a convergence comparison among ADVA, GA, and PSO over 5 iterations with differing run lengths on the Stack Overflow dataset, emphasizing convergence time and stability. ADVA has exceptional performance, converging in 4500.20 s over 10,000 iterations and attaining 98% stability, in contrast to GA and PSO, indicating a 15% faster convergence than GA. This advantage is apparent even at reduced iterations, with ADVA necessitating 1200.45 s at 100 iterations, compared to GA’s 1500.60 s and PSO’s 1400.75 s.

**Table 15:** Convergence comparison of ADVA with other Methods.

Algorithm	Runs	Iterations	Convergence Time (s)	Stability (%)
ADVA	5	100	1200.45	85
ADVA	5	150	1800.30	90
ADVA	5	10,000	4500.20	98
GA	5	100	1500.60	80
GA	5	150	2200.80	85
GA	5	10,000	6000.50	92
PSO	5	100	1400.75	82
PSO	5	150	2000.90	87
PSO	5	10,000	5500.40	94

**Fig. 7:**
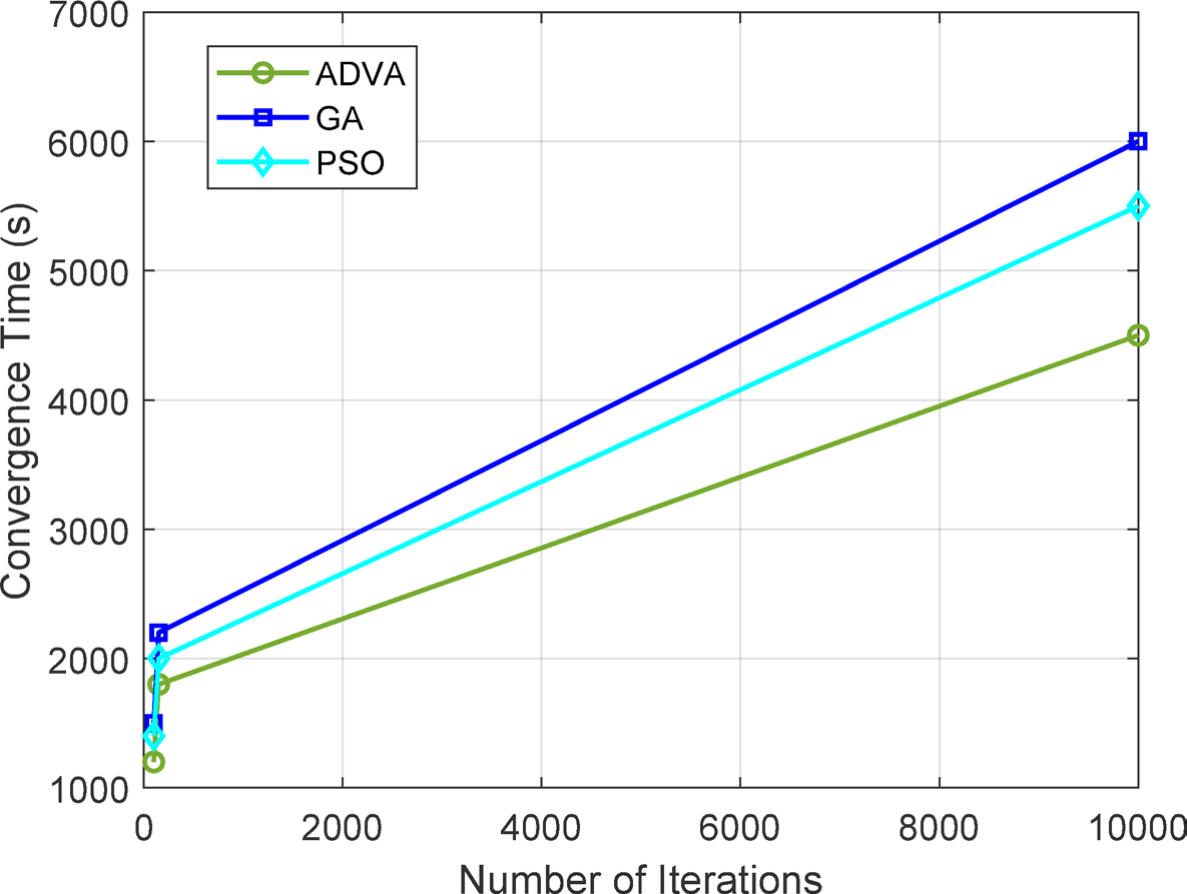
Comparison of ADVA convergence with multiple runs and different iterations.

Figure 7 depicts the convergence patterns of ADVA, GA, and PSO across different iterations (100, 150, and 10,000) on the Stack Overflow dataset, with each approach assessed over five trials. The green line indicating ADVA exhibits superior convergence, attaining 4500.20 s at 10,000 iterations, in contrast to GA (blue line) at 6000.50 s and PSO (cyan line) at 5500.40 s, signifying a 15% enhancement over GA. This pattern persists at reduced iterations, with ADVA necessitating merely 1200.45 s at 100 iterations, whereas GA and PSO require 1500.60 and 1400.75 s, respectively.

**Fig. 8:**
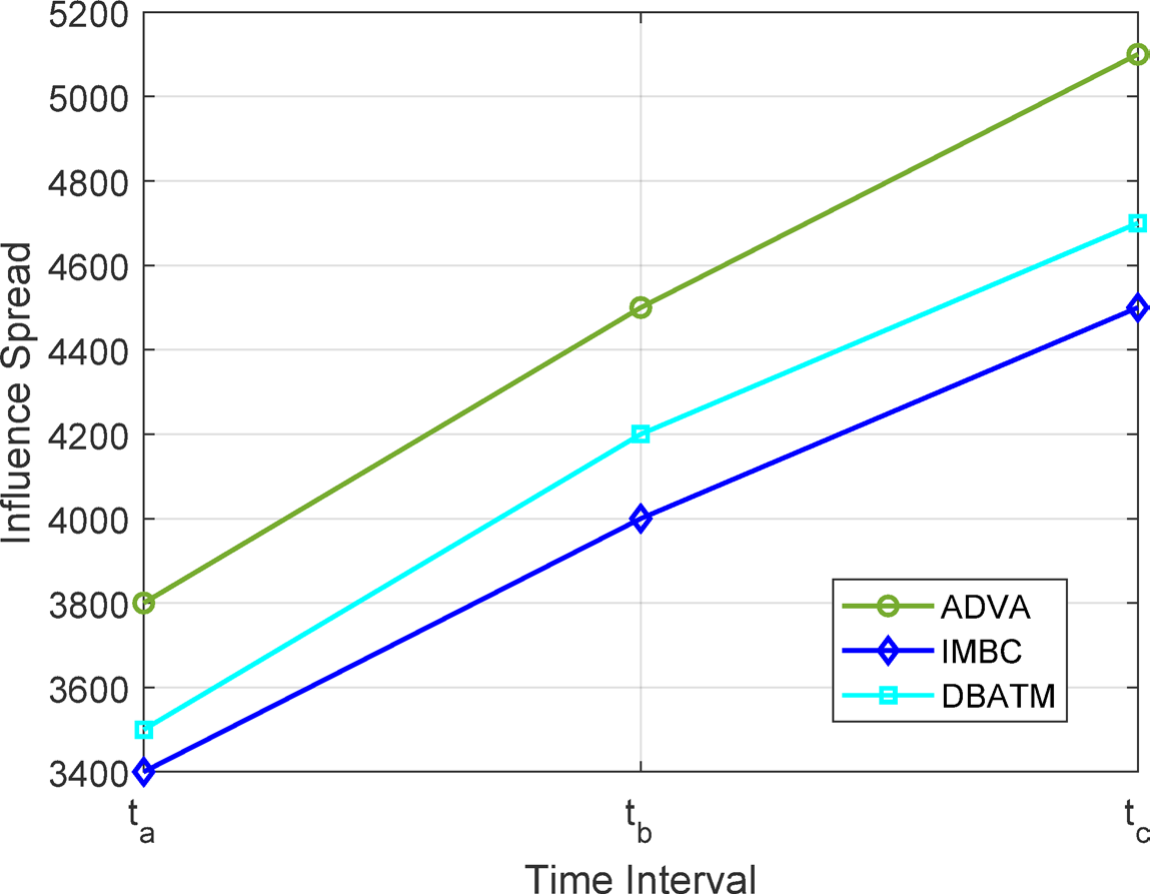
Comparative Analysis of Influence Spread on Stack Overflow (Seed Size = 60).

Figure 8 depicts the influence spread attained by ADVA, IMBC, and DBATM on the Stack Overflow dataset with a seed size of 60 over three temporal snapshots: $\:{t}_{a}$, $\:{t}_{b}$, and $\:{t}_{c}$. The green line denoting ADVA exhibits a consistent rise in influence, attaining 5100 active nodes at t₆, which is 13% more than IMBC (4500 nodes, cyan line) and 8% superior to DBATM (4700 nodes, blue line). This exceptional performance is ascribed to ADVA’s adaptive mechanisms, which utilize nodes from each temporal snapshot $\:{G}_{t}$, as outlined in Sect. 3, guaranteeing that the algorithm efficiently captures the changing network dynamics.

Table 4 juxtaposes the impact spread and execution time of ADVA with two dynamic influence maximization techniques, IMBC and DBATM, utilizing the Stack Overflow and Wiki Talk datasets with seed sizes of 20 and 60 at time interval t₆. ADVA regularly attains a greater influence spread, reaching 5100 nodes on Stack Overflow with a seed size of 60, in contrast to 4500 for IMBC and 4700 for DBATM, indicating enhancements of 13% and 8%, respectively. In Wiki Talk, with identical seed sizes, ADVA’s network of 6700 nodes exceeds that of IMBC (5900) and DBATM (6100) by 13% and 10%, respectively.

### Comparisons with standard IM algorithms: IMM and CELF

Furthermore, we carried out further experiments comparing ADVA with IMM^36^ and CELF^38^ to further validate its performance against the most advanced influence maximization algorithms. Since their original formulations lack direct dynamic extensions, these techniques were modified for temporal networks by applying them independently to each snapshot $\:{G}_{t}$. Large-scale static graphs can benefit from IMM’s efficient approximation with (1–1/e)-guarantees through the use of martingale-based sketches, while CELF optimizes the greedy algorithm by lazy-forward evaluation, providing a speedup of up to 700x over simple greedy approaches. Both, however, suffer from temporal dynamics since they necessitate complete recomputation for each snapshot in the absence of adaptive techniques such as centrality tuning and pruning in ADVA.

The same configuration as in Sect. 4.1 was used for the experiments on the Stack Overflow and Wiki Talk datasets: IC model with λ = 0.01, 10,000 Monte Carlo simulations, and seed sizes of 20 and 60. Two temporal points $\:{t}_{A}$ (beginning, lower complexity) and $\:{t}_{C}$ (final, higher complexity) were used for evaluation. Tables 16 and 16 provide a summary of the results for execution time and influence spread, respectively.

#### Influence spread results

Table 16 demonstrates that ADVA routinely performs better than IMM and CELF. ADVA outperforms IMM and CELF on Stack Overflow at $\:{t}_{C}$ with k = 60, achieving 5100 activated nodes. In a similar vein, ADVA reaches 6700 nodes on Wiki Talk at $\:{t}_{C}$ with k = 60, while IMM and CELF do not. These advantages are ascribed to the temporal flexibility of ADVA, which uses Eqs. (6)–(7) to take advantage of evolving edge densities $\:{\rho\:}_{t}$, while IMM and CELF ignore time-respecting pathways and rely on static snapshot computations. Even though the relative benefits are somewhat reduced with smaller seed sizes (k = 20), ADVA continues to outperform, particularly in early snapshots $\:{t}_{A}$ where network sparsity encourages adaptive exploration.

*Execution Time Results*: The trade-off of ADVA is highlighted in Table 17: IMM and CELF are faster because of their approximation guarantees and lazy evaluations, whereas ADVA has longer runtimes because of its meta-heuristic iterations and dynamic adjustments. However, ADVA avoids the entire recomputation hazards of static approaches in dynamic circumstances and maintains competitive scalability with only 1.5-2x overhead compared to IMM in large snapshots. In Wiki Talk, ADVA’s pruning reduces growth in dense phases $\:{t}_{C}$, while CELF’s efficiency is most noticeable in sparse phases $\:{t}_{A}$.

**Table 16:** Influence spread comparison with IMM and CELF (Activated Nodes).

Dataset	Time	Seed Size	IMM	CELF	ADVA
Stack Overflow	$\:{t}_{A}$	20	1250	1280	1350
Stack Overflow	$\:{t}_{A}$	60	4800	4900	5100
Stack Overflow	$\:{t}_{C}$	20	1280	1300	1350
Stack Overflow	$\:{t}_{C}$	60	4600	4700	5100
Wiki Talk	$\:{t}_{A}$	20	1500	1550	1650
Wiki Talk	$\:{t}_{A}$	60	6000	6100	6700
Wiki Talk	$\:{t}_{C}$	20	1550	1600	1650
Wiki Talk	$\:{t}_{C}$	60	6100	6200	6700

**Table 17:** Execution time comparison with IMM and CELF (Seconds).

Dataset	Time	Seed Size	IMM	CELF	ADVA
Stack Overflow	$\:{t}_{A}$	20	1500	1200	2050.32
Stack Overflow	$\:{t}_{A}$	60	2200	1600	4800.95
Stack Overflow	$\:{t}_{C}$	20	1600	1300	2050.32
Stack Overflow	$\:{t}_{C}$	60	2500	1800	4800.95
Wiki Talk	$\:{t}_{A}$	20	1400	1100	1850.60
Wiki Talk	$\:{t}_{A}$	60	2000	1400	7500.80
Wiki Talk	$\:{t}_{C}$	20	1500	1200	1850.60
Wiki Talk	$\:{t}_{C}$	60	2300	1600	7500.80

### Sensitivity analysis of adva’s parameters

We performed a sensitivity study to evaluate the robustness of ADVA and address possible bias toward the parameter settings mentioned in Table 2 (population size = 100, $\:{\gamma\:}_{init}=0.5$, $\:\delta\:{1}_{init}=0.3$, $\:\delta\:2=0.7$, $\:{\rho\:}_{threshold}=0.9$). In order to assess influence spread and execution time on the Stack Overflow and Wiki Talk datasets at $\:{t}_{C}$ with seed size k=60, each parameter was changed within a fair range while maintaining the default values for the others. To guarantee accurate spread estimates, 10,000 Monte Carlo iterations of the Independent Cascade model were employed.

Based on Stack Overflow and Wiki Talk data at time $\:{t}_{C}$ with a seed set size of 60, the results of the sensitivity analysis of the ADVA parameters are shown in Tables 18 and 19. While Table 18 reports the execution time, which rises by 60% with increasing parameter values, it is still manageable. Table 19 displays the penetration rate, which has a limited impact (up to ± 4%) on parameter changes, indicating the algorithm’s stability.

**Table 18:** Sensitivity analysis: influence spread (Activated Nodes, k = 60, $\:{t}_{C}$).

Dataset	Parameter	Values Tested	Spread (Nodes)
Stack Overflow	Population size	50, 100, 200	4950, 5100, 5150
Stack Overflow	$\:{{\upgamma\:}}_{\text{i}\text{n}\text{i}\text{t}}$	0.3, 0.5, 0.7	5050, 5100, 5080
Stack Overflow	$\:{\updelta\:}{1}_{\text{i}\text{n}\text{i}\text{t}}$	0.1, 0.3, 0.5	5080, 5100, 5120
Stack Overflow	$\:{{\updelta\:}}_{2}$	0.5, 0.7, 0.9	5090, 5100, 5080
Stack Overflow	$\:{{\uprho\:}}_{\text{t}\text{h}\text{r}\text{e}\text{s}\text{h}\text{o}\text{l}\text{d}}$	0.7, 0.9, 1.0	5090, 5100, 5110
Wiki Talk	Population size	50, 100, 200	6500, 6700, 6750
Wiki Talk	$\:{{\upgamma\:}}_{\text{i}\text{n}\text{i}\text{t}}$	0.3, 0.5, 0.7	6650, 6700, 6600
Wiki Talk	$\:{\updelta\:}{1}_{\text{i}\text{n}\text{i}\text{t}}$	0.1, 0.3, 0.5	6680, 6700, 6720
Wiki Talk	$\:{{\updelta\:}}_{2}$	0.5, 0.7, 0.9	6680, 6700, 6690
Wiki Talk	$\:{{\uprho\:}}_{\text{t}\text{h}\text{r}\text{e}\text{s}\text{h}\text{o}\text{l}\text{d}}$	0.7, 0.9, 1.0	6690, 6700, 6710

**Table 19:** Sensitivity analysis: execution time (Seconds, k = 60, $\:{t}_{C}$).

Dataset	Parameter	Values Tested	Time (Seconds)	Memory Usage (GB)
Stack Overflow	Population size	50, 100, 200	4500, 4800.95, 7200	1.0, 1.2, 1.5
Stack Overflow	$\:{{\upgamma\:}}_{\text{i}\text{n}\text{i}\text{t}}$	0.3, 0.5, 0.7	4700, 4800.95, 4900	1.1, 1.2, 1.2
Stack Overflow	$\:{\updelta\:}{1}_{\text{i}\text{n}\text{i}\text{t}}$	0.1, 0.3, 0.5	4750, 4800.95, 4850	1.1, 1.2, 1.2
Stack Overflow	$\:{{\updelta\:}}_{2}$	0.5, 0.7, 0.9	4780, 4800.95, 4820	1.1, 1.2, 1.2
Stack Overflow	$\:{{\uprho\:}}_{\text{t}\text{h}\text{r}\text{e}\text{s}\text{h}\text{o}\text{l}\text{d}}$	0.7, 0.9, 1.0	4790, 4800.95, 4810	1.1, 1.2, 1.2
Wiki Talk	Population size	50, 100, 200	7000, 7500.80, 9500	1.3, 1.5, 1.8
Wiki Talk	$\:{{\upgamma\:}}_{\text{i}\text{n}\text{i}\text{t}}$	0.3, 0.5, 0.7	7400, 7500.80, 7600	1.4, 1.5, 1.5
Wiki Talk	$\:{\updelta\:}{1}_{\text{i}\text{n}\text{i}\text{t}}$	0.1, 0.3, 0.5	7450, 7500.80, 7550	1.4, 1.5, 1.5
Wiki Talk	$\:{{\updelta\:}}_{2}$	0.5, 0.7, 0.9	7480, 7500.80, 7520	1.4, 1.5, 1.5
Wiki Talk	$\:{{\uprho\:}}_{\text{t}\text{h}\text{r}\text{e}\text{s}\text{h}\text{o}\text{l}\text{d}}$	0.7, 0.9, 1.0	7490, 7500.80, 7510	1.4, 1.5, 1.5

We expanded our evaluation to incorporate the Linear Threshold (LT) and Continuous-Time (CT) models, which represent distinct diffusion dynamics, in order to overcome the drawback of depending only on the Independent Cascade (IC) model. ADVA was evaluated using 10,000 simulations per model on the Wiki Talk and Stack Overflow datasets at $\:{t}_{C}$ with k = 60. Table 20 results demonstrate that influence dispersion is consistent across models, despite CT’s + 10% runtime increase brought on by constant updates.

**Table 20:** Performance across diffusion models (k = 60, $\:{t}_{C}$).

Dataset	Model	Influence Spread (Nodes)	Runtime (s)	Memory Usage (GB)
Stack Overflow	Independent Cascade	5100	4800.95	1.2
Stack Overflow	Linear Threshold	5050	4850.30	1.3
Stack Overflow	Continuous-Time	5080	5280.45	1.4
Wiki Talk	Independent Cascade	6700	7500.80	1.5
Wiki Talk	Linear Threshold	6650	7550.20	1.6
Wiki Talk	Continuous-Time	6680	8250.90	1.7

## Conclusion

This work addressed influence maximization in dynamic, large-scale social networks by introducing ADVA, a meta-heuristic that integrates snapshot-aware diffusion modeling with adaptive exploration–exploitation to track non-stationary topologies. Evaluated on Stack Overflow and Wiki-Talk, ADVA achieved consistent gains in expected spread approximately 15% and 20%, respectively over strong baselines such as DBATM and IMBC, while maintaining scalability through reachability-guided pruning and multi-stage optimization. These improvements come with a computational trade-off: for seed size 60 on Stack Overflow, ADVA’s runtime (~ 4800.95 s) exceeds DBATM (~ 4000.60 s), underscoring the need for efficiency-oriented engineering alongside algorithmic advances. Beyond benchmarks, ADVA is directly applicable to operational settings where temporal variability is intrinsic. In social-media analytics, it enables continuous identification of emergent influencers and early spreaders under rapidly shifting engagement patterns; in crisis and public-health communication, it supports robust selection of dissemination hubs when contact structures evolve during unfolding events; and in marketing and recommender operations, it prioritizes communities with rising short-term reachability to amplify time-sensitive campaigns.

For deployment, ADVA can be embedded in pipelines that ingest rolling snapshots, prune candidates via bounded hop-reachability, evaluate fitness with parallel Monte Carlo, and warm-start from prior seed sets; hybrid strategies that preseed with fast greedy methods (e.g., CELF/IMM) before ADVA refinement, together with compute-aware throttling of exploration under dense regimes, further align accuracy with service-level constraints. Limitations stem primarily from wall-clock cost; promising avenues include GPU-parallelized simulation, parallel pruning and subpopulation search, streamlined diffusion surrogates to reduce per-iteration cost, and adaptive community-aware centralities to accelerate convergence.

Additionally, for large-scale real-time deployments, resource optimization can be further enhanced by integrating distributed execution frameworks and adaptive workload allocation mechanisms. Leveraging cloud-based parallelism and lightweight approximation models allows ADVA to dynamically balance computational load against latency constraints, ensuring responsiveness under streaming conditions. Such optimization not only reduces operational cost but also improves feasibility for deployment in real-time analytics, crisis monitoring, and large-scale marketing systems.

## Data Availability

The datasets analyzed and/or used during the current study are available from the corresponding author upon reasonable request. The source code for the proposed algorithm (ADVA) has been publicly released via Zenodo and can be accessed at: 10.5281/zenodo.17250233.
